# Heat stress alters the ovarian proteome in prepubertal gilts

**DOI:** 10.1093/jas/skae053

**Published:** 2024-04-12

**Authors:** Crystal M Roach, Edith J Mayorga, Lance H Baumgard, Jason W Ross, Aileen F Keating

**Affiliations:** Department of Animal Science, Iowa State University, Ames, IA 50011, USA; Department of Animal Science, Iowa State University, Ames, IA 50011, USA; Department of Animal Science, Iowa State University, Ames, IA 50011, USA; Department of Animal Science, Iowa State University, Ames, IA 50011, USA; Department of Animal Science, Iowa State University, Ames, IA 50011, USA

**Keywords:** heat stress, ovary, prepubertal, proteome, pigs

## Abstract

Heat stress (**HS**) occurs when exogenous and metabolic heat accumulation exceeds heat dissipation; a thermal imbalance that compromises female reproduction. This study investigated the hypothesis that HS alters the ovarian proteome and negatively impacts proteins engaged with insulin signaling, inflammation, and ovarian function. Prepubertal gilts (*n* = 19) were assigned to one of three environmental groups: thermal neutral with ad libitum feed intake (**TN**; *n* = 6), thermal neutral pair-fed (**PF**; *n* = 6), or HS (*n* = 7). For 7 d, HS gilts were exposed to 12-h cyclic temperatures of 35.0 ± 0.2 °C and 32.2 ± 0.1 °C, while TN and PF gilts were housed at 21.0 ± 0.1 °C. Liquid chromatography-tandem mass spectrometry (LC-MS/MS) was performed on ovarian protein homogenates. Relative to TN gilts, 178 proteins were altered (*P* ≤* *0.05, log2foldchange* *≥ 1) by HS, with 76 increased and 102 decreased. STRING gene ontology classified and identified 45 biological processes including those associated with chaperone protein refolding, cytoplasmic translational initiation, and immune activation; with a protein–protein interaction web network of 158 nodes and 563 edges connected based on protein function (FDR ≤ 0.05). Relative to PF, HS altered 330 proteins (*P* ≤* *0.05, log2foldchange* *≥ 1), with 151 increased and 179 decreased. Fifty-seven biological pathways associated with protein function and assembly, RNA processing, and metabolic processes were identified, with a protein–protein interaction network of 303 nodes and 1,606 edges. Comparing HS with both the TN and PF treatments, 72 ovarian proteins were consistently altered by HS with 68 nodes and 104 edges, with biological pathways associated with translation and gene expression. This indicates that HS alters the ovarian proteome and multiple biological pathways and systems in prepubertal gilts; changes that potentially contribute to female infertility.

## Teaser

We have identified ovarian molecular changes that are involved in heat stress-induced infertility.

## Introduction

Ambient global temperatures have steadily increased and are predicted to continue to rise (Thornton et al., 2014). Extreme heat events negatively impact infectious and vector-borne diseases (McMichael and Kovats, 2000), impair nutrient absorption (Costello et al., 2009), cause respiratory and cardiovascular illnesses (Robine et al., 2008), and increase human morbidity and mortality (Kovats and Kristie, 2006; Robine et al., 2008; Costello et al., 2009). In addition to human health, heat stress (**HS**) negatively impacts farm animal lactation, growth, and fertility (Baumgard and Rhoads, 2013) and compromises animal health (McMichael, 2013).

HS impairs female reproduction by multiple mechanisms and some of these phenotypic effects include anovulation, reduced conception, and abortion (Love, 1978; Love et al., 1993; Xue et al., 1994). In women, increased ambient temperatures are associated with reduced antral follicle counts (Gaskins et al., 2021) and still-birth (Nyadanu et al., 2022). Interestingly, HS tolerance in female soldiers is related to their menstrual cycle stage; with improved HS tolerance observed during the luteal phase (Yanovich et al., 2019). HS negatively impacts the ovary by altering ovarian steroidogenic enzyme abundance (Nteeba et al., 2015), inducing an ovarian heat shock protein response (Seibert et al., 2019), increasing granulosa cell apoptotic signaling (Luo et al., 2016), and impairing oocyte quality, reducing follicular gonadotropin receptor expression (Ozawa et al., 2005) and gonadotrophin production (Tseng et al., 2006). In addition, HS reduces follicular dominance following recruitment (Ozawa et al., 2005) and increases ovarian autophagy (Hale et al., 2017). Further, HS reduces corpora lutea weight and size (Bidne et al., 2019) and ovarian insulin receptor (INSR) mRNA abundance (Nteeba et al., 2015). Consequently, HS compromises ovarian function and causes infecundity, but a thorough molecular understanding of the etiology is not clear.

This study investigated the hypothesis that the ovarian proteome is sensitive to HS. Liquid chromatography tandem mass spectrometry was utilized to determine ovarian proteomic changes in HS relative to both thermal neutral (**TN**) and pair-fed (**PF**) TN prepubertal gilts. The duration of HS was seven days to facilitate the systemic changes that occur immediately in response to HS, but to avoid eventual acclimation to the heat load, to identify alterations to the ovarian proteome that may be causative for seasonal infertility.

## Material and Methods

### Animal and experimental design

All animal procedures were approved by the Institutional Animal Care and Use Committee at Iowa State University. This study utilized tissues collected from a previously described experiment (Roach et al., 2024). Female crossbred prepubertal gilts were fed a standard diet formulated to meet all nutritional requirements (National Research Council, 2012). Gilts were exposed to constant TN conditions (21.0 ± 0.1 °C, 66.8% relative humidity) or cyclic HS (35.0 ± 0.2 °C from 0700 to 1900 hours, 42.0% relative humidity and 32.2 ± 0.1 °C from 1900 to 0700 hours, 40.7% relative humidity) for 7 d. Environmental temperatures were selected to simulate typical summer conditions in the midwestern region of the United States. All animals were exposed to a 12:12 (L:D)-h cycle throughout the experimental period. The TN gilts were further divided into two subgroups which were either ad libitum fed (TN) or were PF to be a caloric control for the reduction in feed intake (**FI**) that occurred in the HS gilts, and treatments were assigned as thermal neutral (TN; *n* = 6), PF (*n* = 6), or HS (*n* = 7). The PF design controls for the dissimilar effects of FI as malnutrition may also negatively impact female reproduction (Were et al., 2020).

### Tissue collection

Pigs were euthanized on day 7 using a captive bolt and followed by exsanguination and one ovary was immediately collected, weighed, and snap-frozen in liquid nitrogen followed by storage at −80 °C.

### Ovarian protein isolation and quantification

Whole ovarian tissue was powdered with a mortar and pestle on dry ice. Approximately 100 mg of powdered tissue was weighed and lysed by tissue lysis buffer (200 µL; 50 mM Tris-HCl, 1 mM EDTA, pH 8.5) supplemented with Halt protease and phosphatase inhibitor cocktail (P178442, Thermo Scientific, Waltham, MA). Lysed tissue was homogenized by sonication, incubated on ice for 30 min, centrifuged at 10,000 rpm for 15 min at 4 °C and supernatant was collected. The protein concentration was quantified using a Pierce BCA Protein Assay Kit (BCA; 23227, Thermo Scientific) and spectrophotometry detection.

### Liquid chromatography–tandem mass spectrometry

Total ovarian protein samples were prepared as a working solution of 50 µg/µL diluted in lysis buffer. Liquid chromatography-tandem mass spectrometry (LC-MS/MS) analysis was performed as previously described (Clark et al., 2019; González-Alvarez et al., 2021) at The Protein Facility of the Iowa State University Office of Biotechnology. Briefly, 50 µg/µL of total protein (TN: *n* = 6; PF: *n* = 6; HS: *n* = 7) was digested with trypsin/Lys-C for 16 h, dried and reconstituted in buffer A (47.5 µL; 0.1% formic acid/water). The peptide retention time calibration (**PRTC**; 25 fmol/µL) standard was spiked into each sample as an internal control. Protein and PRTC were injected into an LC column and separated by mass spectrometry. Fragmented patterns were compared to MASCOT or Sequest HT theoretical fragmentation patterns for peptide identification. The area of the top three unique peptides per sample was used to identify protein abundance. The PRTC arithmetic mean was used as a normalization factor. The signal intensity was divided by the PRTC arithmetic mean for each peptide. Protein samples identified and detected with three peptide hits were used for analysis. Metaboanalyst 4.0 was used for bioinformatics comparison by the Genome Informatics Facility at Iowa State University. Missing value imputation by Singular Value Decomposition method was performed. Values were filtered based on the interquartile range followed by generalized log transformation. Volcano plots depict alterations to proteins within treatment comparisons. UniProt identified biological, molecular, and pathway information using Kyoto Encyclopedia of Genes and Genomes (KEGG) identifiers for each protein.

### Gene ontology analysis and protein–protein interaction web network

Gene Ontology (**GO**) analysis was conducted using the protein-coding gene classification system Search Tool for the Retrieval of Interacting Genes/Proteins (STRING) and to identify pathways of altered proteins within comparisons. Proteins altered by treatments with a *P* ≤ 0.05 were compared to the *Sus scrofa* reference list for pathway classification. The percentage of each category was calculated by dividing the protein number identified within that category by the total number of altered proteins by a treatment. In addition, ovarian proteins changed by HS when compared to either the TN or PF controls were identified by manual comparison of altered protein lists, and protein–protein interactions/web network was computed using STRING also for that smaller protein subset.

### Statistical analysis

Student’s *t*-test was used to compare treatments with the adjusted *P* value false discovery rate (**FDR**) cutoff of 0.05. A fold change threshold of 1 was used to compare the absolute value of change and expression level between control and treatment values. The threshold for proteins deemed significant in the pathway analysis was *P* ≤ 0.05.

## Results

### Effects of HS exposure on the whole ovarian proteome

**Figure 1.**
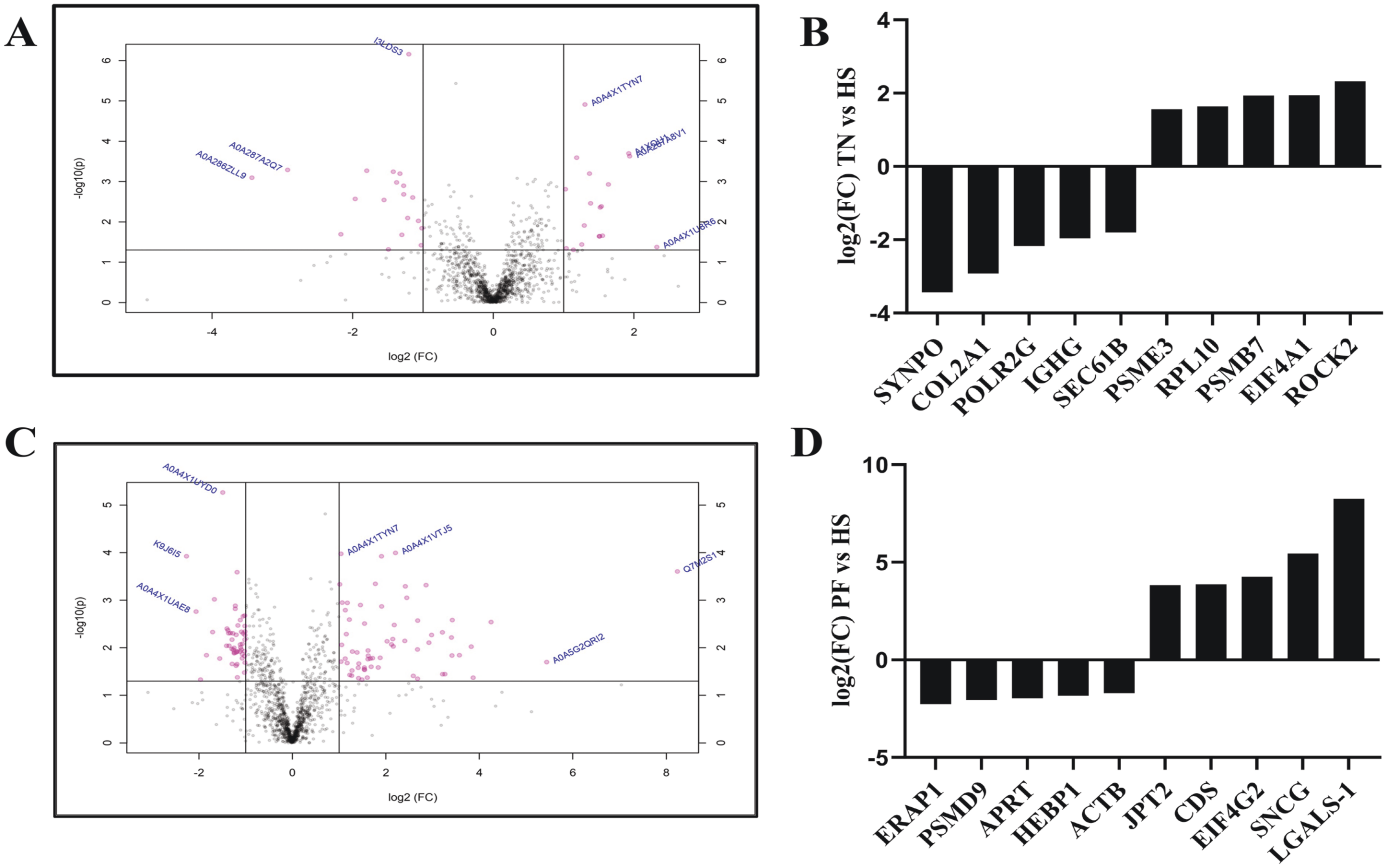
Total ovarian proteins altered by HS. The volcano plots (A and C) illustrate peptides (all dots) identified in pig ovaries. Comparison between (A) TN vs. HS and (C) PF vs. HS. The solid horizontal line indicates where *P* = 0.05, with dots above the line indicating proteins that differ (*P* < 0.05) between treatments and dots below indicating proteins that are not affected by treatment (*P* > 0.05). The solid vertical line indicates log2fold change of < ±1.0 with dots to the right indicating proteins that are increased in abundance and dots to the left denoting proteins that are decreased in their level. The bar charts represent the top five increased and decreased proteins per treatment comparison illustrated as fold-change in (B) TN vs. HS and (D) PF vs. HS in prepubertal gilts. TN: *n* = 6; PF: *n* = 6; HS: *n* = 7; *P* < 0.05.

A total of 2,303, 2,304, and 2,298 proteins were detected in TN, PF, and HS gilt ovaries, respectively. Relative to TN, 178 proteins were altered (*P* ≤ 0.05, log2foldchange ≥ 1; Figure 1A; Supplementary Table S1) by HS, with 76 increased and 102 decreased. Relative to PF gilts, HS altered 330 proteins (*P* ≤ 0.05, log2foldchange ≥ 1; Figure 1C; Supplementary Table S2) with 151 increased and 179 decreased.

The top five ovarian proteins altered by the greatest fold-change that were reduced by HS were SYNPO, COL2A1, POLR2G, IGHG, and SEC61B, and those increased by HS relative to TN control gilts were ROCK2, EIF4A1, PSMB7, RPL10, and PSME3 (P ≤ 0.05; Figure 1B). Relative to PF controls, the top five increased by the greatest fold-change by HS were JPT2, EIF4G2, CDS, SNCG, and LGALS-1, and the top five with the most decreased fold-change were ERAP1, PSMD9, APRT, HEBP1, and ACTB proteins (*P* ≤ 0.05; Figure 1D).

### Functional classification of altered ovarian biological pathways identified by HS

**Figure 2.**
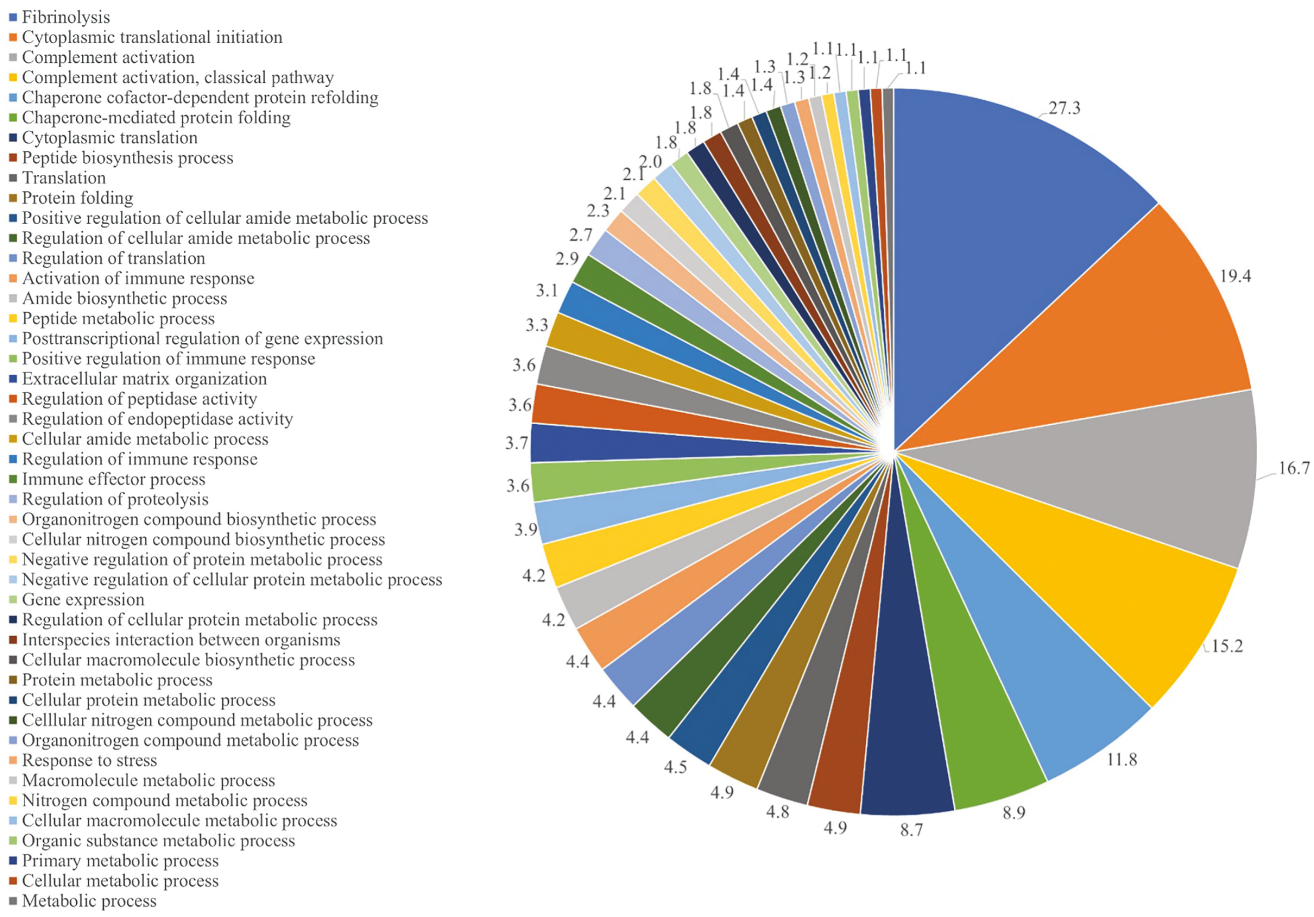
Global distribution of biological processes identified by STRING GO analysis in ovarian proteins altered by HS exposure relative to TN gilts. The pie chart represents the percentage of biological processes altered in TN vs. HS prepubertal gilts; FDR ≤ 0.05. The color code for each pie section is provided to the left of the pie chart.

**Figure 3.**
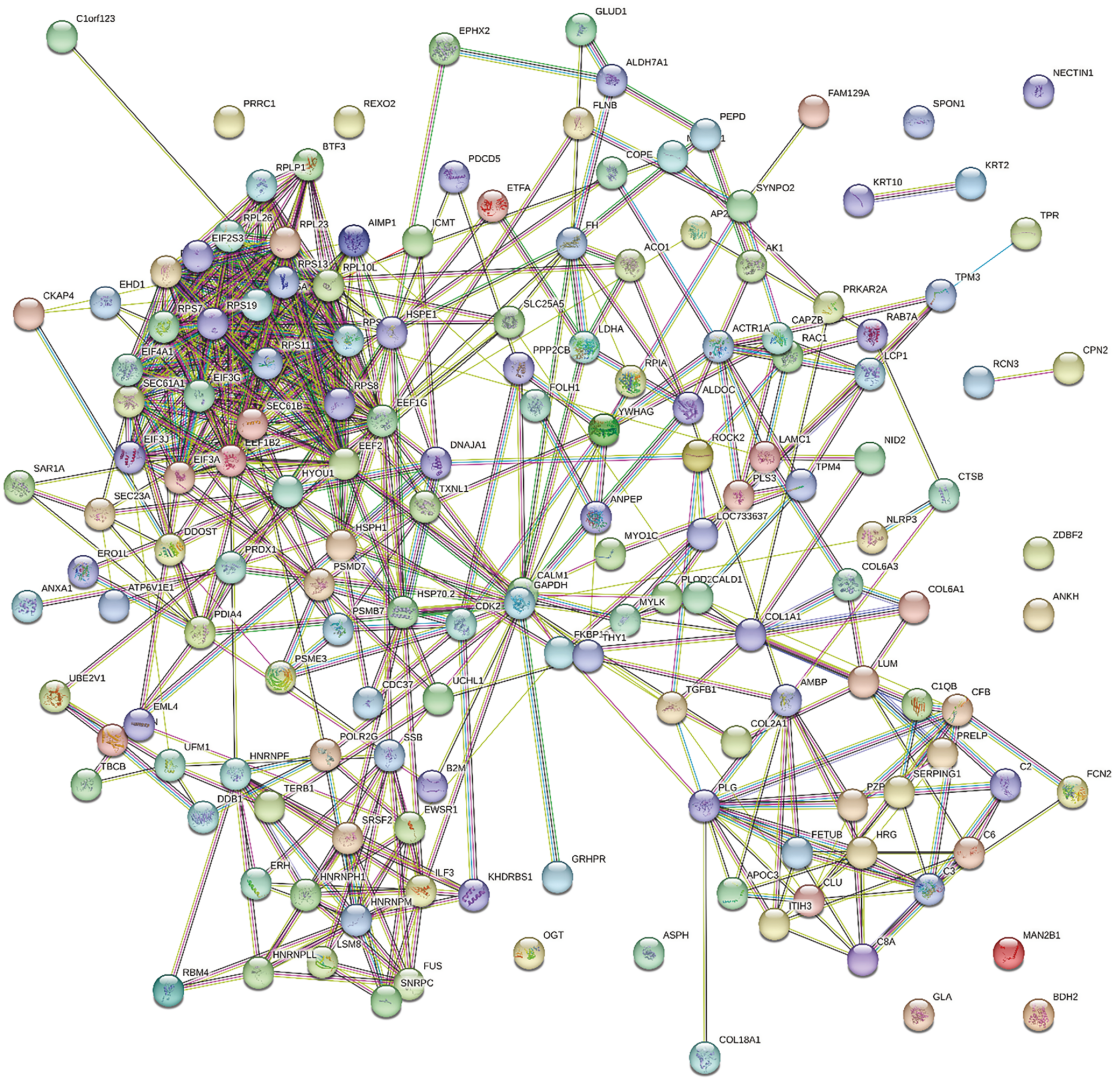
Web network of ovarian proteins altered by HS compared to TN prepubertal gilts by STRING GO analysis. Protein–protein associations of 178 altered ovarian proteins are depicted as a web network. Network nodes represent proteins, with colored nodes indicating the first shell of interactors and white nodes indicating the second shell of interactors. Empty nodes illustrate proteins with unknown 3D structures and filled nodes represent a known or predicted 3D structure. Edges depict protein–protein associations between nodes and illustrate proteins with a shared function. Light blue edges = known interactions curated from databases, light pink edges = experimentally determined known interactions, green edges = gene neighborhood predicted interactions, orange edges = predicted interactions with gene fusions, and navy edges = predicted interactions with gene co-occurrence.

Using STRING GO analysis, 45 biological process pathways were identified. The top biological processes altered by HS relative to TN gilts were associated with fibrinolysis, cytoplasmic translational initiation, complement activation, and chaperone cofactor-dependent protein refolding (FDR ≤ 0.05; Figure 2; Supplementary Table S3). Additionally, the STRING protein–protein interaction network identified 158 nodes and 563 edges associated with protein function that were altered by HS relative to TN gilts (Figure 3).

**Figure 4.**
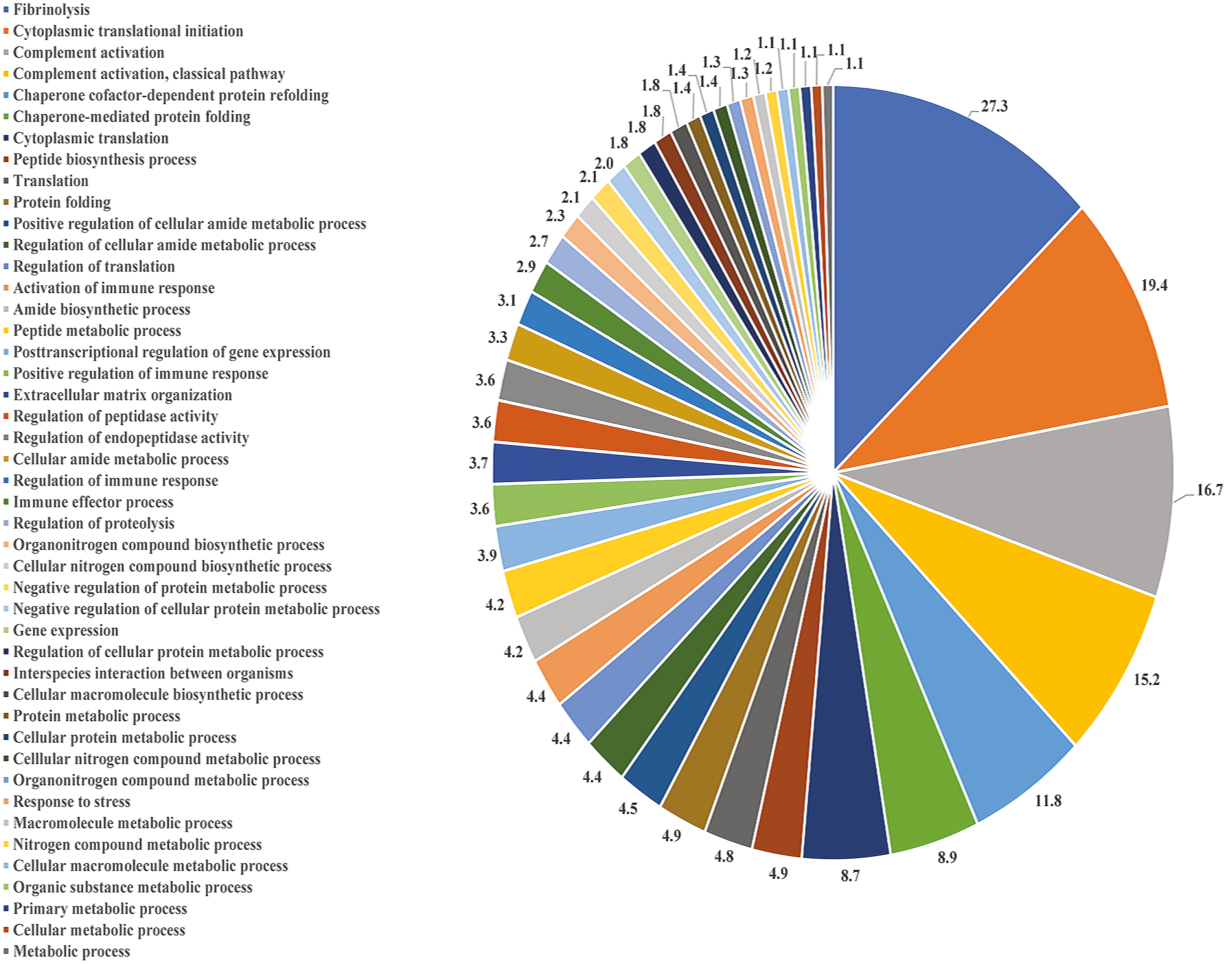
Biological pathway classification of ovarian proteins altered by HS exposure compared to PF females by STRING GO analysis. The pie chart represents the percentage of biological processes altered in the PF vs. HS comparison, FDR ≤ 0.05. The color code for each pie section is provided to the left of the pie chart.

**Figure 5.**
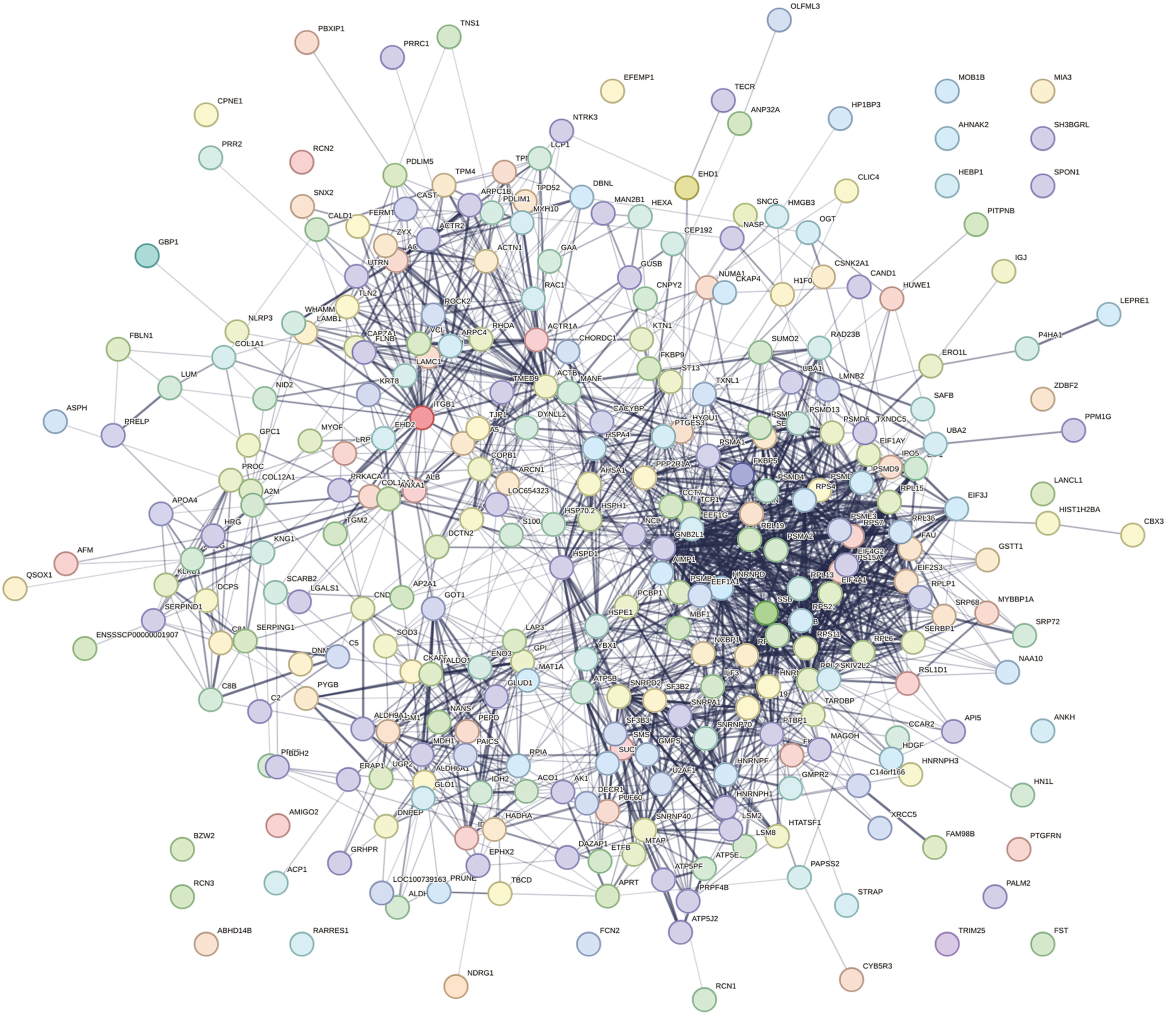
Protein–protein associations of 330 altered ovarian proteins in HS relative to PF gilts depicted as a web network by STRING GO analysis. Network nodes represent proteins, with colored nodes indicating first shell of interactors and white nodes indicating second shell of interactors. Empty nodes illustrate proteins with unknown 3D structure and filled nodes represent a known or predicted 3D structure. Edges depict protein–protein associations between nodes and illustrate proteins with a shared function. Light blue edges = known interactions curated from databases, light pink edges = experimentally determined known interactions, green edges = gene neighborhood predicted interactions, orange edges = predicted interactions with gene fusions, and navy edges = predicted interactions with gene co-occurrence. *P* ≤ 0.05.

STRING identified 57 biological pathways altered by HS relative to PF gilts, with the top five pathways identified as those associated with glyoxylate metabolic process, arp 2/3 complex-mediated actin nucleation, tricarboxylic acid cycle metabolic process, proteasome assembly (23.5%), and chaperone cofactor-dependent protein refolding (14.7%; FDR ≤ 0.05; Figure 4; Supplementary Table S4). Protein–protein interaction network identified 303 nodes and 1,606 edges to be altered by HS relative to PF gilts (Figure 5).

### Identification of common proteins and functions altered by HS relative to TN and PF gilts

**Figure 6.**
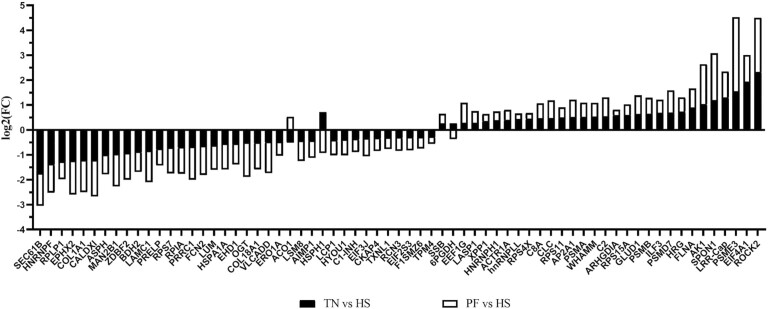
Abundance of ovarian proteins altered by HS regardless of comparison to either TN or PF prepubertal gilts. The bar chart represents the fold-change of ovarian proteins altered by HS compared to (A) TN (black bars) and (B) PF (white bars) prepubertal gilts. TN: *n* = 6; PF: *n* = 6; HS: *n* = 7; *P* ≤ 0.05.

**Figure 7.**
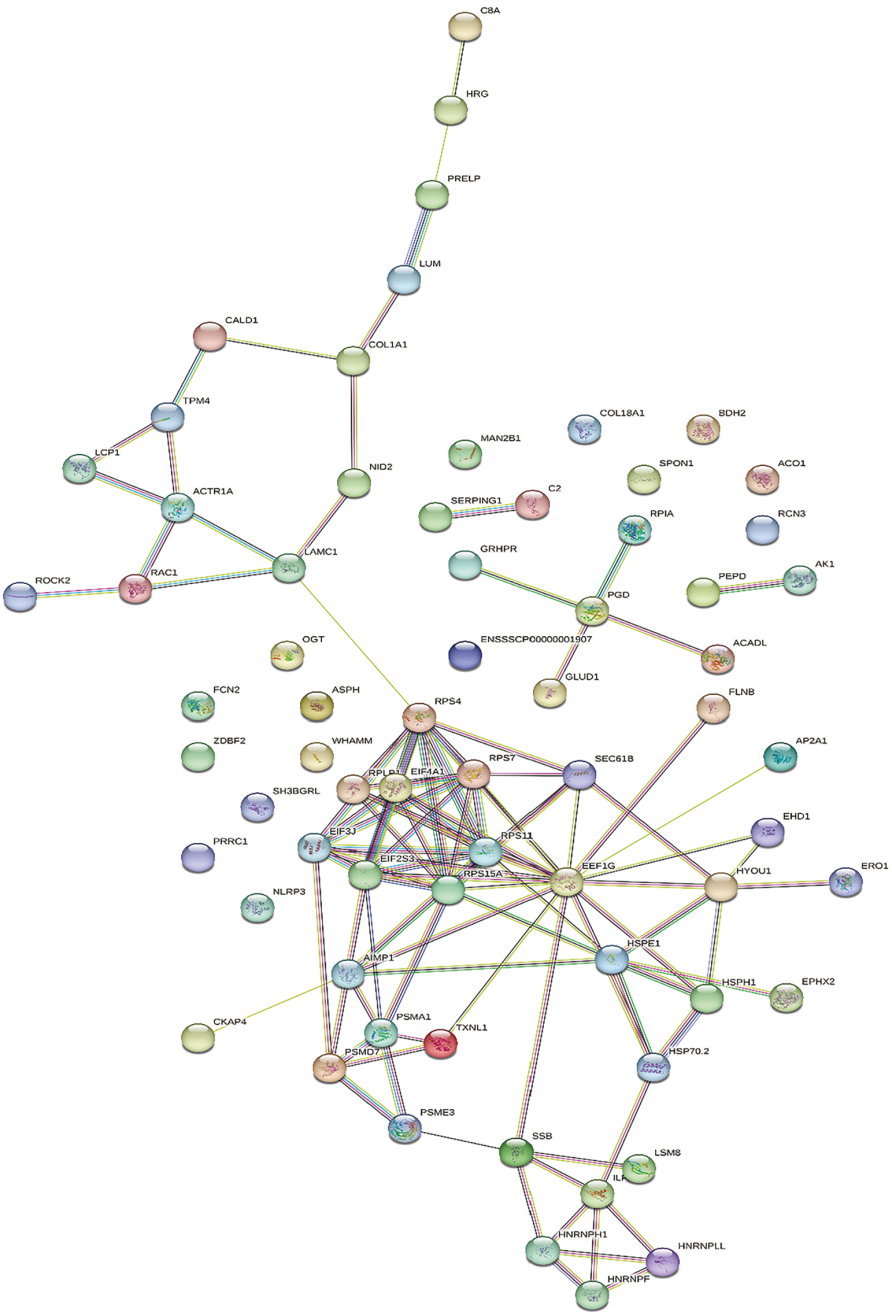
Protein–protein associations of ovarian proteins altered by HS regardless of comparison to either TN or PF prepubertal gilts depicted as a web network by STRING GO analysis. Network nodes represent proteins, with colored nodes indicating first shell of interactors and white nodes indicating second shell of interactors. Empty nodes illustrate proteins with unknown 3D structures and filled nodes represent a known or predicted 3D structure. Edges depict protein-protein associations between nodes and illustrate proteins with a shared function. Light blue edges = known interactions curated from databases, light pink edges = experimentally determined known interactions, green edges = gene neighborhood predicted interactions, orange edges = predicted interactions with gene fusions, navy edges = predicted interactions with gene co-occurrence. *P* < 0.05.

**Figure 8.**
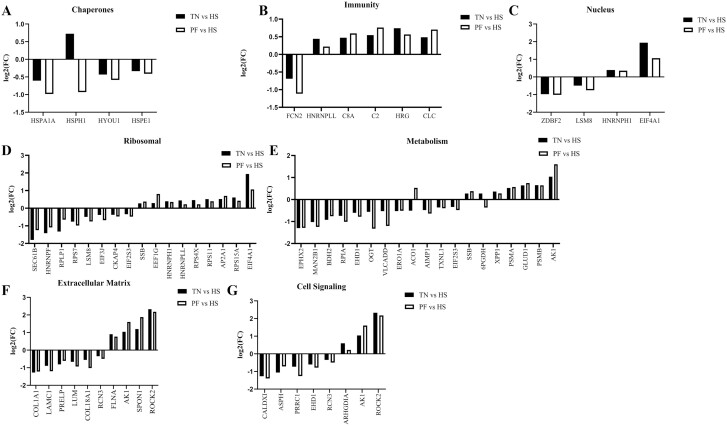
Physiological functions of ovarian proteins altered by HS. The bar charts represent the fold-change of the ovarian proteins that are altered by HS when compared to either TN (black bars) or PF (white bars) prepubertal gilts. Proteins are grouped according to physiological role established by STRING GO analysis: (A) chaperones, (B) immunity, (C) nucleus, (D) ribosomal, (E) metabolism, (F) extracellular matrix, and (G) cell signaling. TN: *n* = 6; PF: *n* = 6; HS: *n* = 7; *P* < 0.05.

As previously mentioned, 178 and 330 ovarian proteins were differentially altered by HS in TN and PF gilts, respectively. Of these proteins, 72 ovarian proteins were consistently altered in HS gilts compared to both TN and PF gilts (Figure 6). Functional protein–protein interactions of this protein subset (*P* ≤ 0.05; Figure 7) depict 67 nodes and 100 edges. STRING identified biological processes from four pathways that were altered by HS (Supplementary Table S5), including chaperone cofactor-dependent protein refolding (11.8%), peptide biosynthetic process (2.4%), translation (2.2%), and gene expression (0.91%). Common proteins are grouped according to functional roles and locations determined by STRING GO analysis (Figure 8) and include chaperones (Figure 8A), immunity (Figure 8B), nuclear (Figure 8C), ribosomal (Figure 8D), metabolic (Figure 8E), extracellular matrix (Figure 8F) and cell signaling (Figure 8G).

## Discussion

HS is a major cause of female infertility (Ross et al., 2017), characterized by increased abortion, smaller litters, delayed puberty, reduced conception rates, and longer wean-to-estrus intervals (Love et al., 1993; Xue et al., 1994; Tast et al., 2002). Prepubertal gilts exposed to acute HS for one week had increased plasma insulin and elevated ovarian steroidogenic acute regulatory protein and CYP19A1 mRNA and protein abundance (Nteeba et al., 2015). Additional HS-induced ovarian impacts in swine include increased autophagy proteins (Hale et al., 2017, 2021), reduced corpora lutea diameter and weight (Bidne et al., 2019), altered insulin-mediated and steroidogenic signaling pathways (Nteeba et al., 2015) and changes to abundance of toll-like 4 receptor (Dickson et al., 2018), estrogen sulfotransferase (Dickson et al., 2018) and heat shock proteins (**HSP**) (Seibert et al., 2019). In women, HS decreases antral follicle counts (Gaskins et al., 2021) and increases the incidence of still-birth (Nyadanu et al., 2022), and evidence suggests that the stage of the menstrual cycle influences the thermal response in soldiers (Yanovich et al., 2019).

Pigs in this study experienced HS conditions that resemble the warm summer months of the Midwestern United States, a period characterized by seasonal infertility. HS causes inappetence (Baumgard and Rhoads, 2013) and malnutrition can also cause infertility (Were et al., 2020; Salem, 2021; Sun et al., 2021), therefore PF controls were included to control for the difference in plane of nutrition. The prepubertal pig was chosen to ensure that the observed effects of HS were not due to the endogenous hormone milieu. Thus, this study aimed to identify ovarian molecular changes in response to HS that may drive negative ovarian outcomes in prepubertal females. These pigs were experiencing HS as evidenced by their increased rectal temperatures and increased respiration rates (Roach et al., 2024).

Ovarian dysfunction results through many mechanisms and can be induced by various stressors. Thus, to evaluate the consequences of HS in prepubertal pigs, whole ovary proteomic profiling using unbiased LC-MS/MS was selected to measure HS-induced altered abundance in proteins compared to both control groups (TN and PF), coupled with additional in silico analysis to predict how affected proteins interact through a protein-to-protein network. Therefore, the remainder of the discussion will focus on the common ovarian proteins altered by HS in both TN groups with functional roles associated with metabolism, immunity, and cellular protein transport.

Nineteen ovarian proteins associated with metabolism were impacted by HS. Of these, HS reduced the abundance of EPHX2, MAN2B1, BDH2, RPIA, EHD1, OGT, VLCADD, ERO1A, AIMP1, TXNL1, EIF2S3 compared to both TN and PF pigs, and increased the abundance of SSB, XPP1, PSMA, GLUD1, PSMB, AK1, and elicited bidirectional effects on ACO1 and 6PGDH compared to the two control groups. Soluble epoxide hydrolase (EPHX2) degrades lipid epoxides to less potent diol metabolites, for conjugation and excretion through xenobiotic metabolism (Newman et al., 2005; Zhan et al., 2021). *EPHX2* mRNA and isoforms are detected in ovarian tissue (Shkolnik et al., 2007) with EPHX2 and EPHX2B isoforms expressed in granulosa cells of preovulatory follicles (Hennebold et al., 2005). Because HS reduces EPHX2 the ovary may be more susceptible to lipid epoxides. Aconitase 1 (ACO1) is an iron-sulfur regulatory protein that plays a role in the TCA cycle (Klausner and Rouault, 1993). HS reduced the ACO1 abundance compared to the TN gilts but increased the ACO1 abundance relative to the PF controls. Reasons for the differential regulation are not clear but suggest an ovarian energetic impact of HS.

Systemic and intestinal inflammation occurs during HS and this drives an immunological response which impacts ovarian cellular proteins. In this study, six immune proteins were altered by HS; increased hnRNPLL, C8A, C2, HRG, CLC, and decreased FCN2. Ficolin-2 (FCN2) is an innate immune pattern recognition protein within the complement system (Garred et al., 2010), and in combination with an increased abundance of the other identified immune response proteins during HS indicates an immune activation response in the ovary. Systemic inflammation occurs during and following HS (Pearce et al., 2013; Ganesan et al., 2017; Mayorga et al., 2020, 2021; Opgenorth et al., 2021; Abeyta et al., 2023; Ruiz-Gonzalez et al., 2023), thus the ovary has an apparent response to systemic inflammation, which is logical since inflammation can be detrimental to fertility (Banerjee et al., 2023; Magata et al., 2023; Shroff, 2023).

Activated HSP maintains cellular proteostasis and has both pro-inflammatory and anti-inflammatory functions. Further, HSP’s maintain intracellular homeostasis through the transportation of damaged proteins to organelles for repair or degradation (Fulda et al., 2010). In this study, the chaperone proteins HSPA1A, HYOU1, the HSPE1 were decreased in response to HS, with a bidirectional effect on HSPH1. Heat shock protein family A (HSP70) member 1A (HSPA1A) and E member 1 (HSPE1 or HSP10) are chaperonins that assist in mitochondrial protein refolding (Bakthisaran et al., 2015; Hartman et al., 1992) during stress. Elevated ovarian HSPA1A abundance has been observed in a previous study in which estrous synchronized pigs were exposed to HS for five days during the follicular phase of estrous cyclicity (Seibert et al, 2019), indicating that HSPA1A is potentially a chaperone biomarker in ovarian tissue during thermal stress regardless of reproductive developmental status. Although proteomic analysis does not provide cell type specificity, previous studies determined *HSP70* presence in granulosa cells and oocytes in pig ovaries (Li et al., 2017), suggesting that damage to oocyte and granulosa cell proteins by HS may ensue, potentially impairing follicle development and oocyte growth prior to puberty. Interestingly, heat shock protein family H (HSP110) member 1 (HSPH1) was bidirectionally altered, with increased ovarian abundance in the HS compared with ad libitum TN gilts but decreased levels when compared to PF gilts. Reasons for this are unclear but, similar to the observations for the ACO1 protein, could be attributable to the differences in energy metabolism, but further research will be needed to determine whether this effect is specific to the reproductive and/or cellular energy status of the female pig. Further, HSPA1A protein abundance decreased in pregnant gilt endometrium due to HS, while HSP family B (small) member 1 (HSPB1) increased due to HS (Adur et al., 2022) so tissue-specific effects may also occur during HS.

In conclusion, ovarian proteins essential for normal function in the prepubertal pig are altered during HS. Unsurprisingly perhaps, HSP responds to a thermal load and are plausible biomarker of the ovarian response to hyperthermia. Future investigations to determine locally where these proteins were altered (i.e., follicle type), the degree of protein activation/inactivation (i.e., posttranslational modification), and their persistence after HS has abated are warranted. Taken together, these findings provide biological foundational knowledge of the ovarian effects of HS.

## Supplementary Material

skae053_suppl_Supplementary_Table_S1

skae053_suppl_Supplementary_Table_S2

skae053_suppl_Supplementary_Table_S3

skae053_suppl_Supplementary_Table_S4

skae053_suppl_Supplementary_Table_S5

## Abbreviations

BCA: bicinchoninic acid
*E2*: 17β-estradiol
FDR: false discovery rate
FI: feed intake
GO: gene ontology
HS: heat stress
HSP: heat shock proteins
KEGG: Kyoto Encyclopedia of Genes and Genomes
LC: liquid chromatography
LC-MS/MS: liquid chromatography-tandem mass spectrometry
LH: luteinizing hormone
PF: pair-fed
PI3K: , phosphatidylinositol-3 kinase
PRTC: peptide retention time calibration
STRING: search tool for the retrieval of interacting genes/proteins
TN: thermal neutral

## CRediT statement

Crystal M. Roach was involved in Investigation, Methodology, Funding Acquisition, Writing—original draft, Formal Analysis, and Data Curation. Edith J. Mayorga was involved in Investigation, Methodology and Writing—original draft; Lance H. Baumgard was involved in Investigation, Methodology, Funding Acquisition, Writing—reviewing and editing; Jason W. Ross was involved in Investigation, Methodology, Funding Acquisition, Writing—reviewing and editing; and Aileen F. Keating was involved in Investigation, Methodology, Funding Acquisition, Formal Analysis, Data Curation, Supervision, and Writing—reviewing and editing
