# Supplementary material for: Heat stress alters the ovarian proteome in prepubertal gilts
Source: J Anim Sci. 2024 Apr 12;102:skae053. doi: 10.1093/jas/skae053 (PMC11025630; doi:10.1093/jas/skae053)
Supplement: skae053_suppl_Supplementary_Table_S1 [file skae053_suppl_supplementary_table_s1.docx]

| **Supplemental Table 1. Comparison of TN vs. HS LC-MS/MS proteomic analysis** | | | | |
| --- | --- | --- | --- | --- |
| **Uniprot ID** | **Protein names** | **Gene names** | **log2(FC)** | ***P* value** |
| A0A286ZLL9 | Synaptopodin | SYNPO | -3.435 | 0.001 |
| A0A287A2Q7 | Collagen type II alpha 1 chain | COL2A1 | -2.927 | 0.001 |
| Q65221 | RNA polymerase II subunit G | POLR2G | -2.169 | 0.020 |
| L8B0U8 | IgG heavy chain | IGHG | -1.965 | 0.003 |
| A0A4X1SH59 | SEC61 translocon subunit beta | SEC61B | -1.801 | 0.001 |
| A0A4X1SUH4 | Bcl-2 associated athanogene | BAG | -1.555 | 0.003 |
| A0A4X1TPV9 | Fructose-bisphosphate aldolase C | ALDOC | -1.493 | 0.048 |
| F1RG16 | Heterogeneous nuclear ribonucleoprotein F | HNRNPF | -1.423 | 0.001 |
| A0A480PNA9 | Solute carrier family 25 member 5 | SLC25A5 | -1.376 | 0.001 |
| A0A4X1T568 | 60S acidic ribosomal protein P1 | RPLP1 | -1.329 | 0.001 |
| A0A4X1VBC2 | Bifunctional epoxide hydrolase 2 | EPHX2 | -1.301 | 0.021 |
| A0A5G2QQE9 | Collagen type I alpha 1 chain | COL1A1 | -1.276 | 0.001 |
| A0A480TAI8 | Caldesmon isoform X1 | CALD1 | -1.275 | 0.002 |
| A0A287BBZ8 | Myosin light chain kinase | MYLK | -1.218 | 0.008 |
| I3LDS3 | Keratin 10 | KRT10 | -1.203 | <0.001 |
| A0A481ARR7 | Alpha-aminoadipic semialdehyde dehydrogenase | ALDH7A1 | -1.147 | 0.002 |
| A0A4X1V1Z6 | Aspartate beta-hydroxylase | ASPH | -1.066 | 0.009 |
| A0A5G2R0E8 | Alpha-mannosidase | MAN2B1 | -1.027 | 0.038 |
| A0A4X1WBN6 | Keratin | KRT | -1.017 | 0.014 |
| A0A4X1V152 | Zinc finger DBF-type domain-containing 2 | ZDBF2 | -0.982 | 0.007 |
| A0A5G2Q7R0 | Collagen type VI alpha 1 chain | COL6A1 | -0.980 | 0.014 |
| A0A480X877 | Transforming growth factor-beta-induced protein | TGFB1 | -0.974 | 0.003 |
| A0A480T5S7 | RAB7A, member RAS oncogene family | RAB7A | -0.959 | 0.006 |
| A0A286ZSA7 | 3-hydroxybutyrate dehydrogenase 2 | BDH2 | -0.926 | 0.004 |
| A0A4X1TV07 | SEC61 translocon alpha 1 subunit | SEC61A1 | -0.912 | 0.013 |
| F1S663 | Laminin subunit gamma 1 | LAMC1 | -0.897 | 0.023 |
| A0A287AGM8 | Dolichyl-diphosphooligosaccharide | DDOST | -0.872 | 0.004 |
| F1S6B4 | Prolargin | PRELP | -0.814 | 0.010 |
| I3L5B2 | 40S ribosomal protein S7 | RPS7 | -0.765 | 0.012 |
| A0A480W9G4 | 40S ribosomal protein S13 | RPS13 | -0.762 | 0.041 |
| A0A481BC81 | Ribose-5-phosphate isomerase isoform X1 | RPIA | -0.745 | 0.016 |
| Q6UAQ9 | Electron transfer flavoprotein alpha subunit, mitochondrial | ETFA | -0.744 | 0.034 |
| A0A4X1V3Z6 | 60S ribosomal protein L9 | RPL9 | -0.743 | 0.050 |
| A0A4X1VSY0 | Myosin regulatory light chain 12B | LOC733637 | -0.739 | 0.035 |
| A0A480W370 | Myosin light chain kinase | MYLK | -0.733 | 0.019 |
| F1RKK5 | Proline rich coiled-coil 1 | PRRC1 | -0.733 | 0.035 |
| A0A4X1WBK5 | Keratin 2 | KRT2 | -0.726 | 0.050 |
| I3LIL4 | Myosin IC | MYO1C | -0.708 | 0.034 |
| A0A4X1SUF1 | Ficolin-2 | FCN2 | -0.691 | 0.010 |
| F1SQ09 | Lumican | LUM | -0.670 | 0.013 |
| A0A2I6SB80 | Thy-1 cell surface antigen | THY1 | -0.663 | 0.044 |
| A0A480P2R0 | Alpha-1-microglobulin/bikunin precursor | AMBP | -0.657 | 0.020 |
| F1SHD6 | Elongation factor 1-beta | EEF1B2 | -0.632 | 0.001 |
| A0A4X1VKL9 | Neuroblast differentiation-associated protein | AHNK | -0.614 | 0.036 |
| A5A8V6 | Heat shock 70kDa protein 1A | HSPA1A | -0.608 | 0.002 |
| F1RQR4 | EH domain containing 1 | EHD1 | -0.605 | 0.014 |
| A0A287AIQ8 | ATPase H+ transporting V1 subunit E1 | ATP6V1E1 | -0.576 | 0.016 |
| Q27HV0 | UDP-N-acetylglucosamine—peptide N-acetylglucosaminyltransferase 110 kDa subunit | OGT | -0.562 | 0.020 |
| A0A480KGD8 | Collagen type VI alpha 3 chain isoform 4 | COL6A3 | -0.562 | 0.045 |
| A0A481C7A2 | Collagen alpha-1 (XVIII) chain | COL18A1 | -0.561 | 0.002 |
| A0A4X1T1R6 | Complement component C6 | C6 | -0.533 | <0.001 |
| A0A480NHK6 | Long-chain specific acyl-CoA dehydrogenase, mitochondrial | ACADL | -0.533 | 0.023 |
| B6CVD7 | Endoplasmic reticulum oxidoreductase 1 alpha | ERO1A | -0.529 | 0.042 |
| A0A4X1TMS8 | Annexin | ANXA1 | -0.516 | 0.028 |
| A0A4X1UMC0 | Aconitate 1, cytoplasmic | ACO1 | -0.508 | 0.007 |
| F1SDC7 | Aldehyde dehydrogenase | ALDH | -0.497 | 0.041 |
| F2Z5P9 | U6 small nuclear RNA associated, LSM8 homolog | LSM8 | -0.494 | 0.044 |
| A0A4X1SUI4 | Ubiquitin-fold modifier 1 | UFM1 | -0.493 | 0.041 |
| A0A287B2P1 | Chloride intracellular channel 1 | CLIC1 | -0.487 | 0.005 |
| A0A4X1UU78 | Aminoacyl tRNA synthetase complex interacting multifunctional protein 1 | AIMP1 | -0.481 | 0.029 |
| A0A4X1W7W4 | Nidogen 2 | NID2 | -0.478 | 0.014 |
| P62831 | 60S ribosomal protein L23 | RPL23 | -0.471 | 0.012 |
| F1RST0 | Heat shock protein family H (Hsp110) member 1 | HSPH1 | -0.466 | 0.015 |
| A0A287ANV7 | Lymphocyte cytosolic protein 1 | LCP1 | -0.461 | 0.009 |
| A0A287BGS6 | Hypoxia up-regulated 1 | HYOU1 | -0.434 | 0.037 |
| A0A5G2R745 | Aminopeptidase N | NPEPPSc | -0.428 | 0.049 |
| A0A480W9F9 | Serpin family G member 1 | SERPING1 | -0.416 | 0.019 |
| A0A4X1VD81 | Eukaryotic translation initiation factor 3 subunit J | EIF3J | -0.386 | 0.042 |
| A0A480UGN8 | Cytoskeleton-associated protein 4 | CKAP4 | -0.376 | 0.023 |
| A0A287BP50 | Thioredoxin like 1 | TXNL1 | -0.361 | 0.013 |
| I3LMU6 | Reticulocalbin 3 | RCN3 | -0.348 | 0.028 |
| A0A4X1V9N4 | Eukaryotic translation initiation factor 2 subunit 3 | EIF2S3 | -0.342 | 0.019 |
| F1SMZ6 | 10 kDa heat shock protein, mitochondrial | HSPE1 | -0.337 | 0.029 |
| D0G7F7 | Tropomyosin 4 | TPM4 | -0.320 | 0.025 |
| A0A480X8T8 | L-lactate dehydrogenase | LDHA | -0.312 | 0.002 |
| F1SAD9 | Protein disulfide-isomerase | PDIA4 | -0.280 | 0.030 |
| Q6QA25 | Tropomyosin 3 | TPM3 | 0.178 | 0.049 |
| A0A4X1UM41 | Tyrosine 3-monooxtgenase/tryptophan 5-monooxygenase activation protein gamma | YWHAG | 0.185 | 0.037 |
| A0A286ZND5 | Peroxiredoxin | PRDX1 | 0.212 | 0.037 |
| A0A4X1VQL9 | Plasminogen | PLG | 0.225 | 0.041 |
| A0A287BL93 | Heterogeneous nuclear ribonucleoprotein M | HNRNPM | 0.250 | 0.010 |
| F1S1V1 | Small RNA binding exonuclease protein factor La | SSB | 0.263 | 0.023 |
| I3L7Y1 | Ribosomal protein L26 | RPL26 | 0.272 | 0.010 |
| A0A480YSA5 | 6-phosphogluconate dehydrogenase, decarboxylating | PGD | 0.273 | 0.031 |
| A0A286ZJ74 | Translocated promoter region, nuclear basket protein | TPR | 0.281 | 0.019 |
| A0A4X1SDD8 | 40s ribosomal protein S19 | RPS19 | 0.284 | 0.003 |
| Q29387 | Elongation factor 1-gamma | EEF1G | 0.288 | 0.012 |
| M3VJZ7 | LIM and SH3 domain protein 1 | LASP1 | 0.289 | 0.007 |
| A0A287AWI9 | Eukaryotic translation elongation factor 2 | EEF2 | 0.293 | 0.029 |
| P00355 | Glyceraldehyde-3-phosphate dehydrogenase | GAPDH | 0.306 | 0.036 |
| A0A5G2RGI4 | Ubiquitin conjugating enzyme E2 N like | UBE2NL | 0.334 | 0.020 |
| A9XFX6 | Capping actin protein of muscle Z-line subunit beta | CAPZB | 0.339 | 0.001 |
| A0A4X1VBU6 | Plastin 3 | PLS3 | 0.360 | 0.018 |
| A0A481BIM5 | Peptidase D | PEPD | 0.361 | 0.004 |
| K7GPT9 | Complement factor B | CFB | 0.368 | 0.024 |
| A0A480TPG6 | Oligoribonuclease, mitochondrial | REXO2 | 0.370 | 0.035 |
| A0A5G2QFP8 | Isoprenylcysteine carboxyl methyltransferase | ICMT | 0.388 | 0.034 |
| A0A287BL05 | Heterogeneous nuclear ribonucleoprotein H1 | HNRNPH1 | 0.396 | 0.010 |
| G8ENL4 | RNA-binding protein FUS | FUS | 0.406 | 0.041 |
| A0A287A059 | Actin related protein 1A | ACTR1A | 0.408 | 0.028 |
| F6Q3L0 | Microtubule associated protein RP/EB family member 1 | MAPRE1 | 0.418 | 0.030 |
| A0A480F5K0 | Heterogeneous nuclear ribonucleoprotein L | HNRNPLL | 0.440 | 0.018 |
| F1SBS4 | Complement C3 | C3 | 0.442 | 0.017 |
| A0A4X1W1U6 | 40S ribosomal protein S4 | RPS4X | 0.456 | 0.031 |
| A0SEH1 | Complement C8A | C8A | 0.471 | 0.009 |
| A0A4X1VQM3 | Clathrin light chain | CLTA | 0.485 | 0.001 |
| A0A4X1TD81 | Glutathione transferase | GSTA4 | 0.488 | 0.024 |
| A0A287APW0 | RNA-binding protein EWS | EWSR1 | 0.488 | 0.026 |
| A0A4X1VTS2 | Ribosomal protein S11 | RPS11 | 0.513 | 0.008 |
| A0A4X1VZ02 | Adaptor protein complex 2 subunit alpha | AP2A1 | 0.519 | 0.024 |
| A0A4X1THY9 | Fumarate hydratase, mitochondrial | FH | 0.520 | 0.018 |
| A0A480SCD0 | Proteasome subunit alpha type | PSMA1 | 0.526 | 0.032 |
| B2CNZ7 | Cathepsin B | CTSB | 0.536 | 0.004 |
| F1S4U9 | EMAP like 4 | EML4 | 0.538 | 0.017 |
| A0A480IPF0 | WASP homolog-associated protein with actin, membranes, and microtubules | WHAMM | 0.539 | 0.037 |
| A0A4X1TPV7 | Rac family small GTPase 1 | RAC1 | 0.540 | 0.016 |
| A0A480Q9T4 | Complement C2 | C2 | 0.547 | 0.016 |
| A0A4X1U3P8 | FKBP prolyl isomerases 10 | FKBP10 | 0.556 | 0.005 |
| A0A4X1VTD3 | Protein phosphatase 2 catalytic subunit beta | PPP2CB | 0.563 | 0.018 |
| C1PIG4 | Protein kinase cAMP-dependent type II regulatory subunit alpha | PRKAR2A | 0.564 | 0.022 |
| A0A480NR20 | Ubiquitin carboxyl-terminal hydrolase L1 | UCHL1 | 0.568 | 0.001 |
| A0A5G2QTD3 | Calmodulin | CALM1 | 0.585 | 0.011 |
| A0A286ZWS0 | SH3 domain-binding glutamic acid-rich-like protein | SH3BGRL | 0.586 | 0.037 |
| E7EI20 | Rho GDP dissociation inhibitor alpha | ARHGDIA | 0.596 | 0.001 |
| A0A4X1VLJ2 | 40S ribosomal protein S15a | RPS15A | 0.606 | 0.009 |
| F2Q9A3 | Peptidyl-prolyl cis-trans isomerase | ppia CYPA | 0.612 | 0.006 |
| A0A4X1UKC2 | Ubiquitin conjugating enzyme E2 variant 1 | UBE2V1 | 0.622 | 0.043 |
| A0A4X1VLC3 | DNA damage-binding protein 1 | DDB1 | 0.628 | 0.012 |
| F1S3I3 | Eukaryotic translation initiation factor 3 subunit G | EIF3G | 0.631 | 0.013 |
| A0A5S8KLN1 | Clusterin | CLU | 0.631 | 0.027 |
| A0A4X1SWC8 | Proteasome 26S subunit, non-ATPase 12 | PSMB12 | 0.636 | 0.036 |
| A0A480TW29 | Serine/arginine-rich splicing factor 2 isoform X1 | SRSF2 | 0.640 | 0.020 |
| A0A4X1VHE8 | Glutamate dehydrogenase 1, mitochondrial | GLUD1 | 0.643 | 0.022 |
| A0A5G2QLU1 | Apolipoprotein C-III | APOC3 | 0.645 | 0.040 |
| A0A481A772 | Proteasome subunit beta | PSMB | 0.651 | 0.010 |
| A0A481D4P9 | Programmed cell death protein 5 | PDCD5 | 0.658 | 0.044 |
| F1SHL3 | SEC23 homolog A, COPII coat complex component | SEC23A | 0.661 | 0.009 |
| A0A4X1VCF5 | Glyoxylate and hydropyruvate reductase | GRHPR | 0.686 | 0.011 |
| A0A4X1U6K8 | COPI coat complex subunit epsilon | COPE | 0.687 | 0.009 |
| F1SE73 | DnaJ homolog subfamily A member 1 isoform 1 | DNAJA1 | 0.687 | 0.029 |
| A0A4X1W3P2 | Galactosidase alpha | GLA | 0.690 | 0.016 |
| K7GL83 | Interleukin enhancer binding factor 3 | ILF3 | 0.694 | 0.034 |
| A0A287AEH0 | Proteasome 26S subunit, non-ATPase 7 | PSMD7 | 0.700 | 0.040 |
| Q8HXL4 | Cytoskeleton-associated protein 1 | CKAP1 | 0.710 | 0.049 |
| A0A481B9A6 | Histidine-rich glycoprotein | HRG | 0.742 | 0.002 |
| A0A287BRF1 | Complement C1q B chain | C1QB | 0.748 | 0.013 |
| A0A480W0S0 | 60S ribosomal protein L21 | PRL21 | 0.752 | 0.012 |
| A0A4X1WB15 | Basic transcription factor 3 | BTF3 | 0.760 | 0.028 |
| Q684M6 | Cell division cycle 37, HSP90 cochaperone | CDC37 | 0.781 | 0.004 |
| A0A480SUZ7 | Fetuin-B isoform 1 | FETUB | 0.782 | 0.002 |
| A0A4X1TZH6 | Enhancer of rudimentary homolog | ERH | 0.786 | 0.001 |
| I3LP11 | KH RNA binding domain containing, signal transduction associated 1 | KHDRBS1 | 0.789 | 0.009 |
| A0A287ARC7 | Cyclin dependent kinase 2 | CDK2 | 0.818 | 0.001 |
| F1S765 | CXXC motif containing zinc binding protein | CZIB | 0.865 | 0.014 |
| A0A480YXA1 | Filamin-B | FLNB | 0.901 | 0.001 |
| K7GRN9 | Niban apoptosis regulator protein 1 | NIBAN1 | 0.916 | 0.029 |
| A0A4X1T8C9 | Carboxypeptidase N subunit 2 | CPN2 | 0.923 | 0.050 |
| A0A4X1TGX3 | RNA binding motif protein 4 | RBM4 | 0.968 | 0.029 |
| F1S4P6 | Eukaryotic translation initiation factor 3 subunit A | EIF3A | 1.026 | 0.002 |
| A0A286ZQ79 | Adenylate kinase isoenzyme 1 | AK1 | 1.038 | 0.045 |
| A0A4X1V8J5 | Synaptopodin | SYNPO2 | 1.139 | 0.049 |
| A0A4X1W1F9 | Nectin cell adhesion molecule 1 | NECTIN1 | 1.186 | <0.001 |
| F1S981 | Spondin 1 | SPON1 | 1.199 | 0.050 |
| Q52NJ3 | Secretion associated RAS related GTPase 1A | SAR1A | 1.258 | 0.036 |
| I3LQ17 | Pregnancy zone protein-like | PZP | 1.268 | 0.050 |
| A0A480KGP4 | Inter-alpha-trypsin inhibitor heavy chain H3 | ITIH3 | 1.294 | 0.012 |
| A0A4X1TYN7 | Leucine-rich repeat | LRR | 1.303 | <0.001 |
| A0A4X1T4T3 | Small nuclear ribonuclearprotein polypeptide C | SNRPC | 1.368 | 0.001 |
| Q00P28 | Beta-2-microglobulin | B2M | 1.384 | 0.003 |
| F1SKE2 | Procollagen-lysine, 2-oxoglutarate 5-dioxygenase 2 | PLOD2 | 1.508 | 0.023 |
| F2Z5F5 | Ribosomal protein S8 | RPS8 | 1.511 | 0.023 |
| A0A4X1W9F5 | Ubiquitin conjugating enzyme E2 M | UBE2M | 1.520 | 0.004 |
| A0A4X1U519 | Adiponectin | ADIPOQ | 1.537 | 0.004 |
| A0A4X1TXU2 | Proteasome activator complex subunit 3 | PSME3 | 1.554 | 0.022 |
| A0A4X1T5U8 | 60S ribosomal protein L10 | RPL10 | 1.637 | 0.001 |
| A1XQU1 | Proteasome subunit beta type-7 | PSMB7 | 1.927 | <0.001 |
| A0A287A8V1 | Eukaryotic translation initiation factor 4A1 | EIF4A1 | 1.940 | <0.001 |
| A0A4X1U8R6 | Rho-associated coiled-coil containing protein kinase 2 | ROCK2 | 2.326 | 0.042 |
