# Supplementary material for: Heat stress alters the ovarian proteome in prepubertal gilts
Source: J Anim Sci. 2024 Apr 12;102:skae053. doi: 10.1093/jas/skae053 (PMC11025630; doi:10.1093/jas/skae053)
Supplement: skae053_suppl_Supplementary_Table_S2 [file skae053_suppl_supplementary_table_s2.docx]

| **Supplemental Table 2. Comparison of PF vs. HS LC-MS/MS proteomic analysis** | | | | |
| --- | --- | --- | --- | --- |
| **UniProtID** | **Protein names** | **Gene names** | **log2(FC)** | ***P* value** |
| K9J6I5 | Aminopeptidase | ERAP1 | -2.266 | <0.001 |
| A0A4X1UAE8 | 26S Proteasome regulatory subunit p27 | PSMD9 | -2.062 | 0.002 |
| A0A4X1TEA2 | Adenine phosphoribosyltransferase | APRT | -1.966 | 0.047 |
| A0A4X1V6D7 | Heme-binding protein 1 | HEBP1 | -1.839 | 0.014 |
| A0A4X1SGG2 | ACTB protein | ACTB | -1.707 | 0.005 |
| D0G0C9 | T-complex protein 1 subunit eta | CCT7 | -1.670 | 0.001 |
| A0A480XZ96 | Aspartate aminotransferase | ASPAT | -1.556 | 0.017 |
| A0A4X1UYD0 | Signal recognition particle subunit 68 | SRP68 | -1.491 | <0.001 |
| A0A480S836 | Glucose-6-phosphate isomerase | GPI | -1.410 | 0.009 |
| A0A480TAI8 | Caldesmon X1 | CALD1 | -1.400 | 0.004 |
| A0A4X1VSE7 | Cytoskeleton-associated protein 5 | CKAP5 | -1.380 | 0.004 |
| A0A4X1SPG7 | N-myc downstream-regulated gene 1 protein | NDRG1 | -1.366 | 0.005 |
| A0A480LN40 | Family with sequence similarity 98 member B | FAM98B | -1.355 | 0.009 |
| Q27HV0 | UDP-N-acetylglucosamine--peptide N-acetylglucosaminyltransferase 110 kDa subunit | OGT | -1.328 | 0.005 |
| A0A4X1TS00 | Signal recognition particle subunit 72 | SRP72 | -1.295 | 0.011 |
| A0A4X1UAA8 | GB1/RHD3-type G domain-containing protein | GBP1 | -1.292 | 0.007 |
| A0A4X1VBC2 | Bifunctional epoxide hydrolase 2 | EPHX2 | -1.290 | 0.022 |
| A0A4X1SD76 | Prune exopolyphosphate 1 | PRUNE1 | -1.274 | 0.005 |
| F1RKK5 | Proline rich coiled-coil 1 | PRRC1 | -1.265 | 0.011 |
| A0A4X1SH59 | Sec61 translocon subunit beta | SEC61B | -1.248 | 0.010 |
| A0A5G2R0E8 | Alpha-mannosidase | MAN2B1 | -1.247 | 0.013 |
| A0A4X1VCP8 | UV excision repair protein | RAD23B | -1.242 | 0.013 |
| I3LAT6 | Isoleucine--tRNA ligase | IARS1 | -1.223 | 0.002 |
| A0A5G2QQE9 | Collagen type I alpha 1 chain | COL1A1 | -1.222 | 0.001 |
| A0A287BHM4 | Apoptosis inhibitor 5 | API5 | -1.206 | 0.013 |
| A0A480NHK6 | Long-chain specific acyl-CoA dehydrogenase, mitochondrial | ACADL | -1.205 | 0.023 |
| F1S663 | Laminin subunit gamma 1 | LAMC1 | -1.203 | 0.005 |
| A0A5G2RN58 | Aldehyde dehydrogenase 1 family member A2 | ALDH1A2 | -1.189 | 0.012 |
| A0A4X1TH18 | Basic leucine zipper and W2 domains 2 | BZW2 | -1.186 | 0.022 |
| I3LUD1 | Superoxide dismutase | SOD3 | -1.184 | 0.012 |
| A0A480TTD3 | Importin-5 isoform X1 | IPO5 | -1.184 | <0.001 |
| A0A4X1V5H0 | Glucogen phosphorylase B | PYGB | -1.177 | 0.042 |
| A0A480PH82 | HIV Tat-specific factor 1 | HTATSF1 | -1.166 | 0.003 |
| A0A286ZY37 | GMP synthase glutamine-hydrolyzing | GMPS | -1.122 | 0.012 |
| A0A4X1SUF1 | Ficolin-2 | FCN2 | -1.116 | 0.009 |
| A0A480LMY7 | Proteasome subunit beta 1 | PSMB1 | -1.115 | 0.003 |
| A0A288CG47 | Proteasome 26S subunit, non-ATPase regulatory subunit 4 | PSMD4 | -1.109 | 0.010 |
| F1RG16 | Heterogeneous nuclear ribonucleoprotein F | HNRNPF | -1.093 | 0.011 |
| A0A4X1V5C3 | Very-long-chain enoyl-CoA reductase | TECR | -1.091 | 0.005 |
| A0A4X1U911 | ATP synthase subunit f, mitochondrial | ATP5J2 | -1.074 | 0.017 |
| A0A5G2QKZ6 | Laminin subunit beta 1 | LAMB1 | -1.073 | 0.014 |
| A0A5K1VDV6 | Actin related protein 2/3 complex subunit 4 | ARPC4 | -1.070 | 0.008 |
| A0A4X1UED9 | Heat shock protein cognate 70 | HSP70 | -1.055 | 0.002 |
| A0A4X1TWE4 | Reticulocalbin-2 | RCN2 | -1.054 | 0.005 |
| A0A4X1VEY3 | Spermine synthase | SMS | -1.026 | 0.033 |
| A0A481C7A2 | Collagen alpha XVIII alpha 1 chain | COL18A1 | -1.024 | 0.012 |
| A0A4X1T7J4 | Acidic nuclear phosphoprotein 32 family member A | ANP32A | -1.024 | 0.015 |
| A0A5G2R6J1 | Proteasome subunit beta 2 | PSMB2 | -1.020 | 0.002 |
| A0A4X1V152 | Zinc finger DBF-type containing 2 | ZDBF2 | -1.018 | 0.007 |
| I3L770 | HECT-type E3 ubiquitin transferase | HUWE1 | -1.018 | 0.003 |
| A0A4X1U7S9 | Nuclear cap binding protein subunit 1 | NCBP1 | -1.015 | 0.021 |
| A0A481BC81 | Ribose-5-phosphate isomerase isoform X1 | RPIA | -1.015 | 0.005 |
| A0A4X1UVY8 | Poly(U)-binding-splicing-factor | PUF60 | -1.014 | 0.005 |
| I3L5B2 | 40S ribosomal protein S7 | RPS7 | -0.986 | 0.001 |
| A5A8V6 | Heat shock 70kDa protein 1A | HSPA1A | -0.985 | 0.001 |
| A0A4X1SPM6 | Casein kinase 2 alpha 1 | CSNK2A1 | -0.985 | 0.029 |
| A0A287BL58 | PBX homeobox interacting protein 1 | PBXIP1 | -0.974 | 0.023 |
| A0A480XI74 | Ribosomal L1 domain-containing protein 1 | RSL1D1 | -0.967 | 0.006 |
| A0A4X1VR00 | Dynamin-1-like protein | DNM1L | -0.955 | 0.048 |
| A0A287BNG8 | PDZ domain-containing protein | AHNAK2 | -0.939 | 0.014 |
| F1SQ09 | Lumican | LUM | -0.933 | <0.001 |
| F1RST0 | Heat shock protein 105 kDa | HSPH1 | -0.931 | <0.001 |
| A0A5G2QR31 | Protein S100 | S100A11 | -0.929 | 0.023 |
| A0A4X1VTJ1 | Lamin B2 | LMNB2 | -0.919 | 0.003 |
| A0A4X1T1L0 | Apolipoprotein A-IV | APOA4 | -0.909 | 0.010 |
| K9J6H8 | Alpha-2-macroglobulin | A2M | -0.893 | 0.001 |
| A0A2C9F3C0 | Beta-glucuronidase | GUSB | -0.884 | 0.022 |
| Q29574 | Histone H2B | Histone H2B | -0.882 | 0.032 |
| A0A481AUZ6 | Zyxin | ZYX | -0.865 | 0.022 |
| A0A4X1T2G4 | Activator of HSP90 ATPase activity 1 | AHSA1 | -0.857 | 0.041 |
| A9YUB1 | Cysteine and histidine-rich domain-containing protein 1 | CHORDC1 | -0.855 | 0.034 |
| A0A4X1SPC1 | Coatomer subunit delta | ARCN1 | -0.855 | 0.026 |
| A0A4X1SRH7 | Glyoxalase I | GLO1 | -0.854 | 0.002 |
| A0A4X1UIC8 | Tumor protein D52 | TPD52 | -0.852 | 0.029 |
| A0A4X1W115 | Small nuclear ribonucleoprotein Sm D2 (Sm-D2) (snRNP core protein D2) | SNRPD2 | -0.850 | 0.016 |
| A0A4X1VQI6 | Scaffold attachment factor B | SAFB | -0.835 | 0.006 |
| A0A4X1UFA1 | Ribosomal protein L19 | RPL19 | -0.832 | 0.003 |
| P10669 | Follistatin | FST | -0.827 | 0.014 |
| H6UWK6 | Olfactomedin like 3 | OLFML3 | -0.820 | 0.002 |
| A0A286ZVS0 | Splicing factor 3B subunit 3 | SF3B3 | -0.816 | 0.031 |
| A0A0B8RTN9 | Glucosidase, alpha | GAA | -0.815 | 0.013 |
| A0A287B283 | LanC like 1 | LANCL1 | -0.801 | 0.029 |
| A0A480U2S8 | U5 small nuclear ribonucleoprotein 40 kDa | SNRNP40 | -0.800 | 0.011 |
| F1RQR4 | EH domain containing 1 | EHD1 | -0.784 | 0.023 |
| D0G780 | N-acetylneuraminic acid synthase | NANS | -0.781 | 0.001 |
| B5APU3 | Actin-related protein 2 | ACTR2 | -0.779 | <0.001 |
| I3LUZ1 | Copine 1 | CPNE1 | -0.764 | 0.010 |
| A0A286ZSA7 | 3-hydroxybutyrate dehydrogenase 2 | BDH2 | -0.762 | 0.015 |
| A0A4X1UYI8 | Collagen type XII alpha 1 chain | COL12A1 | -0.762 | 0.012 |
| A0A287BQW3 | H1 histone family member 0 (Histone H1.0) | H1F0 | -0.758 | 0.019 |
| Q6XGY2 | 2,4-dienoyl-CoA reductase | DECR | -0.757 | 0.019 |
| F2Z5P9 | U6 snRNA-associated Sm-like protein | LSM8 | -0.756 | 0.014 |
| A0A481AUH9 | Low-density lipoprotein receptor-related protein 1 | LRP1 | -0.755 | 0.016 |
| A0A287BLH5 | DEAD-box helicase 5 | DDX5 | -0.747 | 0.001 |
| A0A5G2QCT3 | Fibulin-1 | FBLN1 | -0.738 | 0.002 |
| A0A287AZX9 | Prolyl endopeptidase | PREP | -0.730 | 0.032 |
| I3LR51 | Peptidylprolyl isomerase | FKBP3 | -0.719 | 0.008 |
| I3LD43 | Cytosol aminopeptidase | LAP3 | -0.717 | 0.012 |
| A0A4X1V1Z6 | Aspartate beta-hydroxylase | ASPH | -0.716 | 0.038 |
| F1S3E0 | Transmembrane emp24 domain-containing protein 9 | TMED9 | -0.713 | 0.002 |
| A0A4X1VZ89 | Heterchromatin protein 1-binding protein 3 | HP1BP3 | -0.708 | 0.021 |
| A0A287BBE4 | MYB binding protein 1a | MYBBP1A | -0.704 | 0.028 |
| F2Z5C1 | Annexin A5 | ANXA5 | -0.700 | 0.003 |
| A0A5G2QU20 | Pre-mRNA-processing factor 19 | PRPF19 | -0.698 | 0.003 |
| A0A4X1VUR2 | Polypyrimidine tract-binding protein 1 | PTBP1 | -0.683 | 0.029 |
| A0A4X1W8J3 | TAR DNA-binding protein 43 | TARDBP | -0.681 | 0.023 |
| A0A4X1VD81 | Eukaryotic translation initiation factor 3 subunit J | EIF3J | -0.680 | 0.015 |
| A0A4X1V5Q9 | 3'-phosphoadenosine 5'-phosphosulfate synthase 2 | PAPSS2 | -0.674 | 0.043 |
| A0A4X1VY68 | NADH-cytochrome b5 reductase | CYB5R3 | -0.670 | 0.006 |
| A0A4X1SQU9 | Peptidylprolyl isomerase | FKBP9 | -0.665 | 0.017 |
| P13618 | ATP synthase-coupling factor 6, mitochondrial | ATP5PF | -0.661 | 0.049 |
| A2THZ2 | Albumin | ALB | -0.660 | 0.037 |
| A0A4X1T568 | 60S acidic ribosomal protein 1 | RPLP1 | -0.652 | 0.031 |
| A0A4X1UU78 | Aminoacyl tRNA synthetase complex interacting multifunctional protein 1 | AIMP1 | -0.643 | 0.030 |
| A0A4X1SME4 | Serine/threonine-protein kinase PRP4 homolog | PRPF4B | -0.641 | 0.028 |
| A0A4X1SG10 | Phosphatidylinositol transfer protein beta | PITPNB | -0.638 | 0.021 |
| A0A4X1VAB5 | cAMP-dependent protein kinase catalytic subunit alpha | PRKACA | -0.636 | 0.013 |
| B5APU6 | Actin-related protein 2/3 complex subunit | ARPC1B | -0.632 | 0.037 |
| F1SB63 | T-complex protein 1 subunit alpha | TCP1 | -0.631 | 0.003 |
| A0A286ZKB4 | Complement C5 | C5 | -0.630 | 0.009 |
| A0A480SDM7 | Kinectin isoform X7 | KTN1 | -0.630 | 0.011 |
| F1S6B4 | Prolargin | PRELP | -0.613 | 0.014 |
| A0A4X1UIN8 | Acid phosphatase | ACP1 | -0.602 | 0.027 |
| A0A4X1VKL9 | Neuroblast differentiation-associated protein | AHNK | -0.597 | 0.038 |
| A0A480YMD9 | Prolyl 3-hydroxylase 1 isoform 1 | P3H1 | -0.590 | 0.047 |
| A0A1B2TT55 | Aspartate aminotransferase | GOT1 | -0.588 | 0.018 |
| M3TYC1 | Eukaryotic translation initiation factor 3 subunit B | EIF3B | -0.587 | 0.030 |
| A0A287BGS6 | Hypoxia up-regulated 1 | HYOU1 | -0.585 | 0.014 |
| A0A5G2QFC2 | Malate dehydrogenase | MDH1 | -0.584 | 0.010 |
| A0A5G2RFW9 | Mesencephalic astrocyte derived neutrotrophic factor | MANF | -0.578 | 0.017 |
| A0A5G2RA17 | Aldehyde dehydrogenase 6 family member A1 | ALDH6A1 | -0.574 | 0.026 |
| B5APU4 | Actin-related protein 3 | ARP3 | -0.573 | 0.011 |
| A0A480TMR5 | Aldose 1-epimerase protein | AEP | -0.571 | 0.043 |
| A0A287ANV7 | Lymphocyte cytosolic protein 1 | LCP1 | -0.562 | 0.016 |
| A0A286ZI08 | UTP--glucose-1-phosphate uridylyltransferase | UGP2 | -0.545 | 0.003 |
| A0A286ZUW5 | Procollagen-proline-4-diooxygenase | P4HA1 | -0.537 | 0.007 |
| A0A0B8RSU5 | Hepatoma-derived growth factor | HDGF | -0.535 | 0.030 |
| A0A287BQ81 | Glutathione S-transferase | LOC100739163 | -0.523 | 0.024 |
| A0A4X1TPR9 | T-complex protein subunit epsilon | TCP1 | -0.519 | 0.013 |
| B6CVD7 | ERO1-like protein alpha | ERO1A | -0.512 | 0.024 |
| I3LSD3 | 60S ribosomal protein L13 | RPL13 | -0.507 | 0.022 |
| I3LMU6 | Reticulocalbin 3 | RCN3 | -0.498 | 0.002 |
| Q29554 | Trifunctional enzyme subunit alpha, mitochondrial | HADHA | -0.485 | 0.041 |
| A0A4X1V9N4 | Eukaryotic translation initiation factor 2 subunit 3 | EIF2S3 | -0.483 | 0.039 |
| F1RYT3 | Scavenger receptor class B member 2 | SCARB2 | -0.483 | 0.043 |
| A0A4X1W7W4 | Nidogen 2 | NID2 | -0.481 | 0.024 |
| A0A287AJ85 | U1 small nuclear ribonucleoprotein 70kDa | SNRNP70 | -0.480 | 0.007 |
| A0A480W9F9 | Serpin family G member 1 | SERPING1 | -0.477 | 0.034869 |
| F1S710 | Calcyclin binding protein | CACYBP | -0.477 | 0.005 |
| A0A480UGN8 | Cytoskeleton-associated protein 4 | CKAP4 | -0.474 | 0.034 |
| A0A0B8RZ10 | Ubiquitin-like modifier activating enzyme 1 | UBA1 | -0.466 | 0.004 |
| A0A480HRR5 | Elongation factor thermo unstable, mitochondrial | TUFM | -0.464 | 0.048 |
| F1SJB5 | Annexin A1 | ANXA1 | -0.459 | 0.019 |
| A0A287AM59 | Hexosaminidase subunit alpha | HEXA | -0.456 | 0.031 |
| F1SMZ7 | 60 kDa heat shock protein, mitochondrial | HSP60 | -0.452 | 0.018 |
| A0A480YVB6 | DExD-box helicase 39B | DDX39B | -0.449 | 0.011 |
| A0A4X1W1K9 | EH domain containing 2 | EHD2 | -0.443 | 0.019 |
| B5L0Y6 | Calpastatin | CAST | -0.438 | 0.039 |
| A5A8V8 | LSM8 homolog, U6 small nuclear RNA associated | LSM2 | -0.433 | 0.030 |
| A0A287ADH9 | Chloride intracellular channel protein | CLIC4 | -0.432 | 0.017 |
| A0A0D5BWD2 | Complement C1q binding protein, mitochondrial | C1QBP | -0.421 | 0.045 |
| A0A287A8M1 | Isocitrate dehydrogenase | IDH1 | -0.417 | 0.049 |
| F1SMZ6 | 10 kDa heat shock protein, mitochondrial | HSPE1 | -0.412 | 0.005 |
| Q8MIZ3 | m7GpppX diphosphatase | DCPS | -0.410 | 0.008 |
| A0A481D232 | ATP synthase subunit beta | ATP5B | -0.410 | 0.033 |
| A0A4X1SRF3 | Vinculin | VCL | -0.409 | 0.007 |
| A0A0B8RZZ6 | Transglutaminase 2 | TGM2 | -0.404 | 0.034 |
| A0A287BP50 | Thioredoxin like 1 | TXNL1 | -0.400 | 0.009 |
| A0A5G2QLD8 | Eukaryotic translation initiation factor 1 | EIF1 | -0.389 | 0.022 |
| A0A287BQ72 | Transaldolase | TALDO1 | 0.383 | 0.001 |
| A0A480YSA5 | 6-phosphogluconate dehydrogenase, decarboxylating | PGD | -0.369 | 0.020 |
| A0A4X1SHE8 | Thioredoxin domain-containing protein 5 | TXNDC5 | -0.325 | 0.017 |
| A0A4X1U5R4 | Actinin alpha 1 | ACTN1 | -0.282 | 0.019 |
| A0A481D4T2 | Receptor for activated C kinase 1 | RACK1 | -0.264 | 0.032 |
| A0A480SL93 | Phosphoglucomutase-1 isoform 1 | PGM1 | -0.264 | 0.029 |
| I3LJ87 | 40S Ribosomal protein S2 | RPS2 | -0.255 | 0.043 |
| D0G7F7 | Tropomyosin 4 | TPM4 | -0.241 | 0.047 |
| I3LEC2 | Poly(rC) binding protein 1 | PCBP1 | -0.239 | 0.027 |
| A0A4X1UY89 | Heat shock protein family (70 kDa) protein 4 | HSPA4 | -0.188 | 0.048 |
| E7EI20 | Rho GDP dissociation inhibitor alpha | ARHGDIA | 0.218 | 0.017 |
| A0A480F5K0 | Heterogeneous nuclear ribonucleoprotein L | HNRNPL | 0.222 | 0.032 |
| A0A4X1W1U6 | 40S ribosomal protein S4 | RPS4X | 0.222 | 0.035 |
| A0A480V2R5 | 26S proteasome non-ATPase regulatory subunit 6 | PSMD6 | 0.230 | 0.018 |
| Q0Z8U2 | 40S ribosomal protein S3 | RPS3 | 0.242 | 0.048 |
| A0A481BIM5 | Peptidase D | PEPD | 0.280 | 0.036 |
| A0A5G2R0H0 | Electron transfer flavoprotein subunit beta | ETFB | 0.285 | 0.041 |
| A0A480L3S8 | Myosin heavy chain 10 | MYH10 | 0.294 | 0.013 |
| A0A5G2QZY6 | GTP-binding nuclear protein Ran | RAN | 0.320 | 0.051 |
| A0A4X1USV7 | Enolase | ENO3 | 0.321 | 0.043 |
| A0A480VHU8 | FAU ubiquitin-like and ribosomal protein S30 | FAU | 0.333 | 0.029 |
| A0A287BL05 | Heterogeneous nuclear ribonucleoprotein H1 | HNRNPH1 | 0.351 | 0.008 |
| A0A286ZL65 | Ribosomal protein L15 | RPL15 | 0.352 | 0.008 |
| A0A4X1TI48 | Nucleolin | NCL | 0.352 | 0.015 |
| A0A287AEC1 | MOB kinase activator 1B | MOB1B | 0.369 | 0.016 |
| F1S1V1 | Small RNA binding exonuclease protection factor | SSB | 0.379 | 0.004 |
| A0A4X1SKB8 | Dynein light chain | DYNLL2 | 0.384 | 0.011 |
| A0A4X1U2W5 | Carosine dipeptidase 2 | CNDP2 | 0.390 | 0.044 |
| Q2YGT9 | 60S ribosomal protein L6 | RPL6 | 0.393 | <0.001 |
| A0A4X1VTS2 | 40S ribosomal protein S11 | RPS11 | 0.395 | 0.050 |
| A0A4X1TKK7 | Eukaryotic translation initiation factor 4C | EIF1AY | 0.395 | 0.039 |
| A0A287A059 | Actin related protein 1A | ACTR1A | 0.398 | 0.019 |
| A0A4X1VLJ2 | 40S ribosomal protein S15a | RPS15A | 0.424 | 0.004 |
| A0A480K1P6 | Filamin-A isoform 1 | FLNA | 0.433 | 0.035 |
| F1SFF4 | RNA transcription, translation and transport factor protein | RTRAF | 0.441 | 0.002 |
| F1SFI7 | Alpha-2-HS-glycoprotein | AHSG | 0.456 | 0.010 |
| F1S982 | Coatomer subunit beta | COPB1 | 0.460 | 0.026 |
| M3VJZ7 | LIM and SH3 domain protein 1 | LASP1 | 0.468 | 0.004 |
| A0A480JJD1 | 60S ribosomal protein L27 | RPL27 | 0.474 | 0.025 |
| A0A5G2QNW1 | SERPINE1 mRNA binding protein 1 | SERBP1 | 0.486 | 0.017 |
| A0A480Z819 | Eukaryotic translation initiation factor 3 subunit C | EIF3C | 0.487 | 0.045 |
| A0A480XE38 | Reticulocalbin-1 | RCN1 | 0.490 | 0.014 |
| F1RVC9 | Heterogeneous nuclear ribonucleoprotein D | HNRNPD | 0.496 | 0.017 |
| P33198 | Isocitrate dehydrogenase, mitochondrial | IDH2 | 0.508 | 0.009 |
| F1RMA3 | Cell cycle and apotosis regulator 2 | CCAR2 | 0.522 | 0.004 |
| K7GL83 | Interleukin enhancer binding factor 3 | ILF3 | 0.524 | 0.020 |
| A0A4X1UMC0 | Aconitate, cytoplasmic | ACO1 | 0.529 | 0.006 |
| A0A4X1W9B8 | Canopy FGF signaling regulator 2 | CNPY2 | 0.533 | 0.049 |
| A0A480IPF0 | WASP homolog-associated protein with actin, membranes and microtubules | WHAMM | 0.549 | 0.011 |
| A0SEH2 | Complement component C8B | C8B | 0.560 | 0.042 |
| A0A287A1G4 | PDZ and LIM domain 5 | PDLIM5 | 0.563 | 0.004 |
| A0A4X1WC51 | S-methyl-5'-thioadenosine phosphorylase | MTAP | 0.563 | 0.014 |
| A0A481B9A6 | Histidine-rich glycoprotein | HRG | 0.566 | 0.011 |
| A0A480SCD0 | Proteasome subunit alpha type 1 | PSMA1 | 0.568 | 0.013 |
| A0A480Y2E3 | Kininogen-1 isoform 1 | KNG1 | 0.586 | 0.026 |
| A0SEH1 | Complement component C8A | C8A | 0.595 | 0.042 |
| A0A287A4Y2 | GMP reductase (GMPR) | GMPR2 | 0.604 | 0.022 |
| F1SAY0 | Prostaglandin F2 receptor inhibitor | PTGFRN | 0.607 | 0.035 |
| I3LFV4 | Y-box binding protein 1 | YBX1 | 0.615 | 0.003 |
| A0A480K0D5 | Cullin-associated and neddylation-dissociated 1 | CAND1 | 0.643 | 0.011 |
| A0A481A772 | Proteasome subunit beta | PSMB | 0.644 | 0.010 |
| A0A4X1W070 | S-adenosylmethionine synthase | SAMS | 0.652 | 0.008 |
| A0A4X1TPV7 | Rac family small GTPase 1 | RAC1 | 0.665 | 0.008 |
| B1PSB6 | Adiponectin | ADIPOQ | 0.673 | 0.012 |
| A0A287ADG1 | Drebrin like | DBNL | 0.680 | 0.035 |
| F1S232 | 4-trimethylaminobutyraldehyde dehydrogenase | ALDH9A1 | 0.692 | 0.006 |
| A0A4X1TPG2 | Protein phosphatase, Mg2+/Mn2+ dependent 1G | PPM1G | 0.694 | 0.044 |
| A0A4X1VZ02 | Adaptor protein complex 2 subunit alpha | AP2A1 | 0.700 | 0.015 |
| F1RUM1 | Afamin | AFM | 0.701 | 0.032 |
| A0A4X1VQM3 | Clathrin light chain | CLTA | 0.702 | <0.001 |
| A0A4X1W1F9 | Nectin cell adhesion molecule 2 | NECTIN2 | 0.703 | 0.006 |
| A0A287BP49 | Nuclear mitotic apparatus protein 1 | NUMA1 | 0.709 | 0.032 |
| A0A480VS42 | Nuclear autoantigenic sperm protein isoform 2 | NASP | 0.735 | 0.007 |
| A0A288CG57 | Elongation factor 1-alpha | EEF1A1 | 0.741 | 0.002 |
| A0A4X1VHE8 | Glutamate dehydrogenase 1, mitochondrial | GLUD1 | 0.747 | 0.007 |
| A0A480Q9T4 | Complement C2 | C2 | 0.762 | 0.020 |
| A0A480YXA1 | Filamin-B 2 | FLNB | 0.766 | 0.002 |
| A0A480JX12 | Integrin beta | ITGB1 | 0.778 | 0.018 |
| A0A287A2S6 | SH3 domain binding glutamate rich protein like | SH3BGRL | 0.793 | 0.003 |
| A0A4X1SGN5 | Retinoic acid receptor responder 1 | RARRES1 | 0.801 | 0.046 |
| F1RJX8 | Coatomer subunit alpha | COPA | 0.802 | 0.003 |
| Q29387 | Elongation factor 1-gamma | EEF1G | 0.806 | <0.001 |
| A0A4X1TX13 | Talin 2 | TLN2 | 0.815 | 0.019 |
| A0A4X1SMT6 | Vitamin K-dependent protein C | PROC | 0.823 | 0.019 |
| I3LAB6 | Proteasome subunit alpha type 2 | PSMA2 | 0.836 | 0.001 |
| A0A4X1TMS3 | MIA SH3 domain ER export factor 3 | MIA3 | 0.859 | 0.025 |
| A0A4X1U1L3 | Ig-like domain-containing protein | - | 0.862 | 0.021 |
| A0A287AEH0 | Proteasome 26S subunit, non-ATPase 7 | PSMD7 | 0.882 | 0.028 |
| A0A5K1TZ27 | PDZ and LIM domain protein 1 | PDLIM1 | 0.885 | 0.013 |
| A0A4X1W3Y3 | Mago-bind domain-containing protein | MAGO | 0.888 | 0.013 |
| A0A4X1TI02 | Glutathione S-transferase | GSTA4 | 0.891 | 0.019 |
| A0A480EV23 | Calpain-1 catalytic subunit | CAPN1 | 0.894 | 0.018 |
| A0A4X1UBD2 | Tight junction protein 1 | TJP1 | 0.929 | 0.025 |
| A0A4X1SK13 | Ig-like domain-containing protein | - | 0.964 | 0.001 |
| F1S682 | Sulfhydryl oxidase (EC 1.8.3.2) | QSOX1 | 0.973 | 0.026 |
| F2Z5W4 | Heterogeneous nuclear ribonucleoprotein H3 | HNRNPH3 | 0.993 | 0.002 |
| A0A4X1SLB9 | MTR4 exosome RNA helicase | MTREX | 1.013 | <0.001 |
| A0A4X1TYN7 | Leucine rich repeat | LRR | 1.040 | <0.001 |
| A0A5G2QJK4 | Small ubiquitin-related modifier (SUMO) | SUMO2 | 1.041 | 0.020 |
| A0A4X1TGG9 | Theronine tRNA ligase | TARS1 | 1.054 | 0.009 |
| A0A287A8V1 | Eukaryotic translation initiation factor 4A1 | EIF4A1 | 1.065 | 0.001 |
| A0A480WXA4 | PALM2-AKAP2 protein isoform 2 | PALM2-AKAP2 | 1.130 | 0.017 |
| A0A5G2R0B7 | Small nuclear ribonucleoprotein polypeptide A | SNRPA1 | 1.132 | 0.002 |
| A0A287BMK7 | Sorting nexin 2 | SNX2 | 1.154 | 0.005 |
| A0A287B0T7 | Chromobox protein homolog 3 isoform X1 | CBX3 | 1.172 | 0.021 |
| A0A4X1VCF5 | Glyoxylate and hydroxypyruvate reductase | GRHPR | 1.176 | 0.001 |
| A0A287A4Y3 | Ig-like domain-containing protein | - | 1.219 | 0.003 |
| A0A287AJB4 | Splicing factor U2AF 35 kDa subunit | U2AF1 | 1.227 | 0.037 |
| A0A4X1VWY2 | Kallikrein | KLK3 | 1.269 | 0.038 |
| A0A5G2R557 | Utrophin | UTRN | 1.276 | 0.030 |
| A0A481BLI9 | Serpin family D member 1 | SERPIND1 | 1.283 | 0.012 |
| A0A4X1WBI1 | Keratin 8 | KRT8 | 1.383 | 0.013 |
| A0A480Z0X7 | Splicing factor 3B subunit 2 | SF3B2 | 1.408 | 0.026 |
| A0A4X1U5P7 | Ras-related protein Rab-18 | RAB18 | 1.415 | 0.044 |
| A0A287AV98 | Proteasome 26S subunit, non-ATPase 11 | PSMD11 | 1.421 | 0.022 |
| A0A4X1ST20 | Ras homolog family member A | RHOA | 1.456 | 0.001 |
| A0A287AD53 | Fermitin family member 2 | FERMT2 | 1.489 | 0.047 |
| K7GRT4 | N(alpha)-acetyltransferase 10, NatA catalytic subunit | NAA10 | 1.537 | 0.028 |
| P54612 | Protein phosphatase 2A 65 kDa regulatory subunit A alpha isoform | PPP2R1A | 1.540 | 0.026 |
| A0A4X1UVD0 | Myoferlin | MYOF | 1.543 | 0.003 |
| F1SQL2 | EGF containing fibulin extracellular matrix protein 1 | EFEMP1 | 1.545 | 0.029 |
| A0A286ZQ79 | Adenylate kinase isoenzyme 1 | AK1 | 1.602 | 0.042 |
| A0A4X1SNR4 | Ig-like domain-containing protein | - | 1.616 | 0.017 |
| A0A4X1UM40 | Tensin 1 | TNS1 | 1.619 | 0.011 |
| A0A4X1VZI2 | 26S proteasome non-ATPase regulatory subunit 13 | PSMD13 | 1.623 | 0.015 |
| A0A287ARA3 | SUMO-activating enzyme subunit 2 | UBA2 | 1.666 | 0.017 |
| P53590 | Succinate--CoA ligase [GDP-forming] subunit beta, mitochondrial | SUCLG2 | 1.685 | 0.025 |
| A0A286ZQD3 | FK506 binding protein 5 | FKBP5 | 1.716 | 0.017 |
| A0A287BKR0 | Cortactin | CTTN | 1.775 | <0.001 |
| A0PFK6 | F-actin-capping protein subunit alpha | CAPZA1 | 1.837 | 0.026 |
| F1S981 | Spondin 1 | SPON1 | 1.878 | 0.016 |
| A0A287ATH4 | Glypican 1 | GPC1 | 1.906 | <0.001 |
| A0A480QWI4 | Histone H1.4 | Histone H1.4 | 1.909 | 0.001 |
| A0A4X1VRZ2 | 60S ribosomal protein L36 | RPL36 | 2.025 | 0.007 |
| A0A287BRY0 | Tropomyosin alpha-1 chain | TPM1 | 2.121 | 0.009 |
| A0A5G2QTM2 | Prostaglandin E synthase 3 | PTGES3 | 2.147 | 0.007 |
| A0A4X1SEA1 | ABhydrolase-1 domain-containing 14B | ABHD14B | 2.159 | 0.009 |
| A0A4X1U8R6 | Rho-associated coiled-coil containing protein kinase 2 | ROCK2 | 2.179 | 0.003 |
| A0A4X1VTJ5 | DAZ associated protein 1 | DAZAP1 | 2.205 | <0.001 |
| A0A4X1W6J2 | Dynatic subunit 2 | DCTN2 | 2.405 | 0.007 |
| A0A287BJW3 | High mobility group protein B3 | HMGB3 | 2.416 | 0.001 |
| A0A480NH74 | Mitogen-activated protein kinase | MAPK | 2.447 | 0.001 |
| A0A480YLE4 | Serine-threonine kinase receptor-associated protein | STRAP | 2.586 | 0.039 |
| A0A4X1TK83 | Ig-like domain-containing protein | - | 2.676 | 0.011 |
| A0A4X1VTD1 | Centrosome protein 192 | CEP192 | 2.678 | 0.045 |
| A0A480IKI4 | Tubulin-specific chaperone D | TBCD | 2.681 | 0.003 |
| A0A4X1TR60 | ATP synthase F1 subunit epsilon | ATP5F1E | 2.860 | <0.001 |
| A0A286ZQE9 | Death associated protein | DAP | 2.921 | 0.008 |
| A0A4X1TXU2 | Proteasome activator complex subunit 3 | PSME3 | 2.978 | 0.005 |
| A0A287BQC8 | Joining chain of multimeric IgA and IgM | JCHAIN | 3.210 | 0.005 |
| A0A4X1UQ16 | X ray repair cross complementing 5 | XRCC5 | 3.213 | 0.036 |
| A0A4X1T5Y1 | Glycine tRNA ligase | GARS1 | 3.264 | 0.036 |
| A0A4X1TRK4 | AIRc domain-containing protein | PAICSa | 3.403 | 0.006 |
| A0A4X1SKD0 | FKBP prolyl isomerase 1A | FKBP1A | 3.414 | 0.015 |
| A0A4X1SHM4 | Tripartite motif containing 25 | TRIM25 | 3.422 | 0.003 |
| A0A4X1SE35 | Endothelial differentiation related factor 1 | EDF1 | 3.573 | 0.014 |
| A0A5G2QK57 | Jupiter microtubule associated homolog 2 | JPT2 | 3.827 | 0.009 |
| A0A4X1TQS8 | CDP-diacylglycerol synthase | CDS | 3.868 | 0.043 |
| A0A4X1TU60 | Eukaryotic translation initiation factor 4 gamma 2 | EIF4G2 | 4.251 | 0.003 |
| A0A5G2QRI2 | Gamma-synuclein | SNCG | 5.441 | 0.020 |
| Q7M2S1 | Galectin-1 | LGALS-1 | 8.238 | <0.001 |
