# Supplementary material for: Heat stress alters the ovarian proteome in prepubertal gilts
Source: J Anim Sci. 2024 Apr 12;102:skae053. doi: 10.1093/jas/skae053 (PMC11025630; doi:10.1093/jas/skae053)
Supplement: skae053_suppl_Supplementary_Table_S3 [file skae053_suppl_supplementary_table_s3.docx]

| **Supplemental Table 3. STRING Gene Ontology - Biological Process Classification for TN vs HS comparison** | | | | | |
| --- | --- | --- | --- | --- | --- |
| **Functional Classification** | **Number of hits** | **Total # of genes** | **%** | **Strength** | **FDR** |
| Fibrinolysis | 3 | 11 | 27.3 | 1.57 | 0.032 |
| Cytoplasmic translational initiation | 6 | 31 | 19.4 | 1.42 | <0.001 |
| Complement activation | 8 | 48 | 16.7 | 1.36 | <0.001 |
| Complement activation, classical pathway | 5 | 33 | 15.2 | 1.32 | 0.003 |
| Chaperone cofactor-dependent protein refolding | 4 | 34 | 11.8 | 1.21 | 0.039 |
| Chaperone-mediated protein folding | 5 | 56 | 8.9 | 1.09 | 0.022 |
| Cytoplasmic translation | 9 | 103 | 8.7 | 1.08 | <0.001 |
| Peptide biosynthesis process | 23 | 466 | 4.9 | 0.83 | <0.001 |
| Translation | 22 | 454 | 4.8 | 0.82 | <0.001 |
| Protein folding | 8 | 164 | 4.9 | 0.82 | 0.011 |
| Positive regulation of cellular amide metabolic process | 7 | 155 | 4.5 | 0.79 | 0.041 |
| Regulation of cellular amide metabolic process | 21 | 480 | 4.4 | 0.78 | <0.001 |
| Regulation of translation | 19 | 432 | 4.4 | 0.78 | <0.001 |
| Activation of immune response | 9 | 203 | 4.4 | 0.78 | 0.008 |
| Amide biosynthetic process | 24 | 574 | 4.2 | 0.76 | <0.001 |
| Peptide metabolic process | 24 | 574 | 4.2 | 0.76 | <0.001 |
| Posttranscriptional regulation of gene expression | 21 | 534 | 3.9 | 0.73 | <0.001 |
| Positive regulation of immune response | 14 | 384 | 3.6 | 0.7 | <0.001 |
| Extracellular matrix organization | 9 | 246 | 3.7 | 0.7 | 0.028 |
| Regulation of peptidase activity | 13 | 359 | 3.6 | 0.69 | 0.001 |
| Regulation of endopeptidase activity | 12 | 336 | 3.6 | 0.69 | 0.004 |
| Cellular amide metabolic process | 26 | 796 | 3.3 | 0.65 | <0.001 |
| Regulation of immune response | 17 | 549 | 3.1 | 0.63 | <0.001 |
| Immune effector process | 13 | 447 | 2.9 | 0.6 | 0.01 |
| Regulation of proteolysis | 17 | 623 | 2.7 | 0.57 | 0.002 |
| Organonitrogen compound biosynthetic process | 30 | 1312 | 2.3 | 0.49 | <0.001 |
| Cellular nitrogen compound biosynthetic process | 28 | 1318 | 2.1 | 0.46 | <0.001 |
| Negative regulation of protein metabolic process | 21 | 986 | 2.1 | 0.46 | 0.005 |
| Negative regulation of cellular protein metabolic process | 19 | 942 | 2.0 | 0.44 | 0.02 |
| Gene expression | 36 | 1976 | 1.8 | 0.4 | <0.001 |
| Regulation of cellular protein metabolic process | 46 | 2539 | 1.8 | 0.39 | <0.001 |
| Interspecies interaction between organisms | 20 | 1121 | 1.8 | 0.39 | 0.048 |
| Cellular macromolecule biosynthetic process | 25 | 1427 | 1.8 | 0.38 | 0.014 |
| Protein metabolic process | 62 | 4301 | 1.4 | 0.29 | <0.001 |
| Cellular protein metabolic process | 52 | 3672 | 1.4 | 0.29 | <0.001 |
| Celllular nitrogen compound metabolic process | 45 | 3245 | 1.4 | 0.28 | 0.004 |
| Organonitrogen compound metabolic process | 70 | 5194 | 1.3 | 0.27 | <0.001 |
| Response to stress | 38 | 2851 | 1.3 | 0.26 | 0.039 |
| Macromolecule metabolic process | 76 | 6318 | 1.2 | 0.22 | <0.001 |
| Nitrogen compound metabolic process | 80 | 6916 | 1.2 | 0.2 | <0.001 |
| Cellular macromolecule metabolic process | 56 | 4931 | 1.1 | 0.19 | 0.048 |
| Organic substance metabolic process | 90 | 8037 | 1.1 | 0.18 | <0.001 |
| Primary metabolic process | 84 | 7531 | 1.1 | 0.18 | <0.001 |
| Cellular metabolic process | 83 | 7616 | 1.1 | 0.17 | 0.003 |
| Metabolic process | 93 | 8727 | 1.1 | 0.16 | 0.001 |
