# Supplementary material for: Heat stress alters the ovarian proteome in prepubertal gilts
Source: J Anim Sci. 2024 Apr 12;102:skae053. doi: 10.1093/jas/skae053 (PMC11025630; doi:10.1093/jas/skae053)
Supplement: skae053_suppl_Supplementary_Table_S4 [file skae053_suppl_supplementary_table_s4.docx]

| **Supplemental Table 4. STRING Gene Ontology - Biological Process Classification for PF vs HS comparison** | | | | | |
| --- | --- | --- | --- | --- | --- |
| **Functional Classification** | **Number of hits** | **Total # of genes** | **%** | **Strength** | **FDR** |
| Glyoxylate metabolic process | 3 | 7 | 42.9 | 1.48 | 0.047 |
| Arp 2/3 complex-mediated actin nucleation | 5 | 19 | 26.3 | 1.27 | 0.006 |
| Tricarboxylic acid metabolic process | 4 | 16 | 25.0 | 1.25 | 0.031 |
| Proteasome assembly | 4 | 17 | 23.5 | 1.22 | 0.035 |
| Chaperone cofactor-dependent protein refolding | 5 | 34 | 14.7 | 1.02 | 0.037 |
| Purine ribonucleoside monophosphate metabolic process | 5 | 34 | 14.7 | 1.02 | 0.037 |
| Chaperone-mediated protein folding | 8 | 56 | 14.3 | 1.01 | 0.001 |
| Negative regulation of coagulation | 5 | 35 | 14.3 | 1.01 | 0.04 |
| Cellular aldehyde metabolic process | 6 | 43 | 14.0 | 1 | 0.015 |
| Tricarboxylic acid cycle | 5 | 37 | 13.5 | 0.98 | 0.047 |
| Peptidyl-proline modification | 7 | 63 | 11.1 | 0.9 | 0.015 |
| Protein folding | 16 | 164 | 9.8 | 0.84 | <0.001 |
| Regulation of RNA splicing | 13 | 158 | 8.2 | 0.77 | <0.001 |
| Blood coagulation | 9 | 119 | 7.6 | 0.73 | 0.019 |
| Regulation of mRNA splicing, via spliceosome | 8 | 112 | 7.1 | 0.71 | 0.047 |
| mRNA splicing, via spliceosome | 16 | 243 | 6.6 | 0.67 | <0.001 |
| RNA splicing | 26 | 395 | 6.6 | 0.67 | <0.001 |
| Purine ribonucleotide biosynthetic process | 9 | 138 | 6.5 | 0.67 | 0.04 |
| Purine-containing compound biosynthetic process | 10 | 157 | 6.4 | 0.66 | 0.023 |
| Ribonucleoprotein complex assembly | 11 | 196 | 5.6 | 0.6 | 0.033 |
| Positive regulation of protein-containing complex assembly | 11 | 198 | 5.6 | 0.6 | 0.035 |
| Purine ribonucleotide metabolic process | 14 | 262 | 5.3 | 0.58 | 0.01 |
| Generation of precursor metabolites and energy | 15 | 290 | 5.2 | 0.57 | 0.008 |
| mRNA processing | 26 | 504 | 5.2 | 0.57 | <0.001 |
| Peptide biosynthetic process | 24 | 466 | 5.2 | 0.56 | <0.001 |
| Wound healing | 12 | 234 | 5.1 | 0.56 | 0.035 |
| Translation | 23 | 454 | 5.1 | 0.56 | <0.001 |
| Peptide metabolic process | 28 | 574 | 4.9 | 0.54 | <0.001 |
| Purine-containing compound metabolic process | 15 | 315 | 4.8 | 0.53 | 0.016 |
| Regulation of translation | 19 | 432 | 4.4 | 0.5 | <0.001 |
| mRNA metabolic process | 28 | 648 | 4.3 | 0.49 | <0.001 |
| Actin cytoskeleton organization | 24 | 569 | 4.2 | 0.48 | 0.002 |
| Regulation of peptidase activity | 15 | 359 | 4.2 | 0.47 | 0.044 |
| Regulation of cellular amide metabolic process | 20 | 480 | 4.2 | 0.47 | 0.008 |
| Carbohydrate metabolic process | 18 | 455 | 4.0 | 0.45 | 0.023 |
| Supramolecular fiber organization | 20 | 508 | 3.9 | 0.45 | 0.015 |
| Nucleobase-containing small molecule metabolic process | 17 | 447 | 3.8 | 0.43 | 0.047 |
| Cellular amide metabolic process | 30 | 796 | 3.8 | 0.43 | 0.001 |
| Cellular protein-containing complex assembly | 27 | 735 | 3.7 | 0.42 | 0.004 |
| Positive regulation of organelle organization | 19 | 535 | 3.6 | 0.4 | 0.047 |
| Protein-containing complex assembly | 37 | 1087 | 3.4 | 0.38 | <0.001 |
| RNA processing | 31 | 924 | 3.4 | 0.38 | 0.004 |
| Gene Expression | 59 | 1976 | 3.0 | 0.33 | <0.001 |
| Organonitrogen compound biosynthetic process | 39 | 1312 | 3.0 | 0.33 | 0.005 |
| Oxidation-reduction process | 30 | 1029 | 2.9 | 0.32 | 0.035 |
| Cellular nitrogen compound biosynthetic process | 38 | 1318 | 2.9 | 0.31 | 0.009 |
| Small molecule metabolic process | 42 | 1541 | 2.7 | 0.29 | 0.011 |
| Cellular nitrogen compound metabolic process | 83 | 3245 | 2.6 | 0.26 | <0.001 |
| Cellular component assembly | 54 | 2144 | 2.5 | 0.25 | 0.008 |
| Cellular component biogenesis | 60 | 2412 | 2.5 | 0.25 | 0.004 |
| Organonitrogen compound metabolic process | 109 | 5194 | 2.1 | 0.17 | 0.001 |
| Primary metabolic process | 155 | 7531 | 2.1 | 0.17 | <0.001 |
| Organic substance metabolic process | 164 | 8037 | 2.0 | 0.16 | <0.001 |
| Nitrogen compound metabolic process | 141 | 6916 | 2.0 | 0.16 | <0.001 |
| Cellular metabolic process | 150 | 7616 | 2.0 | 0.15 | <0.001 |
| Metabolic process | 170 | 8727 | 1.9 | 0.14 | <0.001 |
| Macromolecule metabolic process | 120 | 6318 | 1.9 | 0.13 | 0.012 |
