# Supplementary material for: Heat stress alters the ovarian proteome in prepubertal gilts
Source: J Anim Sci. 2024 Apr 12;102:skae053. doi: 10.1093/jas/skae053 (PMC11025630; doi:10.1093/jas/skae053)
Supplement: skae053_suppl_Supplementary_Table_S5 [file skae053_suppl_supplementary_table_s5.docx]

| **Supplemental Table 4. STRING Gene Ontology - Biological Process Classification for Common Proteins between TN and PF Gilts Exposed to HS** | | | | | |
| --- | --- | --- | --- | --- | --- |
| **Functional Classification** | **Number of hits** | **Total # of genes** | **%** | **Strength** | **FDR** |
| Chaperone cofactor-dependent protein refolding | 4 | 34 | 11.8 | 1.58 | 0.0130 |
| Peptide biosynthetic process | 11 | 466 | 2.4 | 0.88 | 0.0025 |
| Translation | 10 | 454 | 2.2 | 0.85 | 0.0087 |
| Gene Expression | 18 | 1976 | 0.91 | 0.47 | 0.0342 |
